# Comprehensive Phytochemical Profiling and Multi‐Target Biological Evaluation of *Hibiscus sabdariffa* L. Calyx Extracts From Libya: A Promising Functional Food Candidate With Antibacterial, Antioxidant, Anti‐Inflammatory, and Cytotoxic Properties: Validation Through In Silico Approaches

**DOI:** 10.1002/fsn3.72056

**Published:** 2026-06-30

**Authors:** Ahmed Saeed Kabbashi, Zuhir S. Mussa Akrim, Mohammed B. Suliman, Sanadelaslam S. A. El‐Hddad, Ahmed Ali Mustafa, Maryam Mohammed Ibrahim, Esraa Radwan Ibrahim, Mona Rafea Mosa, Rama Burhan Hasan

**Affiliations:** ^1^ Department of Biomedical Science, Faculty of Pharmacy Omar Al‐Mukhtar University Al‐Bayda Libya; ^2^ Department of Microbiology and Parasitology Medicinal and Aromatic Plants and Traditional Medicine Research Institute, National Centre for Research Khartoum Sudan; ^3^ Department of Microbiology, Faculty of Medical Laboratory Sciences International University of Africa Khartoum Sudan; ^4^ Department of Pharmacology and Toxicology, Faculty of Pharmacy Omar Al‐Mukhtar University Al‐Bayda Libya; ^5^ Department of Pharmaceutical Chemistry, Faculty of Pharmacy Omar Al‐Mukhtar University Al‐Bayda Libya; ^6^ Department of Botany and Microbiology, Faculty of Science University of Gezira Wad Madani Sudan

**Keywords:** ADMET, antibacterial, anti‐inflammatory, antioxidant, cytotoxicity, functional food, GC–MS, *Hibiscus sabdariffa*, molecular docking

## Abstract

The global escalation of antimicrobial resistance necessitates the exploration of plant‐based therapeutics with multi‐target mechanisms. This study provides a comprehensive evaluation of the Libyan *Hibiscus sabdariffa* L. calyx extract, encompassing phytochemical characterization, in vitro bioactivity, and in silico validation. Aqueous and ethanolic extracts were prepared and screened for antibacterial activity against clinically relevant multidrug‐resistant isolates (including determination of minimum inhibitory concentration (MIC) and minimum bactericidal concentration (MBC)), antioxidant capacity via the 2,2‐diphenyl‐1‐picrylhydrazyl (DPPH) radical assay, anti‐inflammatory effects by measuring their inhibition of albumin denaturation, and cytotoxicity using the brine shrimp lethality model. Gas Chromatography–Mass Spectrometry (GC–MS) analysis of the ethanolic extract revealed that the predominant fatty acid esters were oleic acid methyl ester (41.01%) and palmitic acid methyl ester (28.76%). Both extracts exhibited broad‐spectrum antibacterial activity, with MIC values ranging from 6.25 to 25 mg/mL and MBC/MIC ratios ≤ 2, confirming their bactericidal activity. The aqueous and ethanolic extracts showed superior efficacy against *Pseudomonas aeruginosa* (20.0 ± 1.73 mm inhibition zone; MIC: 6.25 mg/mL) and *Staphylococcus aureus* (20.0 ± 1.00 mm; MIC: 6.25 mg/mL), respectively. The antioxidant activities were comparable (IC_50_: 283.6 vs. 292.1 μg/mL). Remarkably, the aqueous extract demonstrated potent anti‐inflammatory activity (IC_50_: 129.81 μg/mL) and showed low preliminary toxicity in the brine shrimp lethality assay (LD_50_ > 1000 μg/mL), whereas the ethanolic extract exhibited moderate toxicity (LD_50_: 345.5 μg/mL). Preliminary phytochemical screening of the aqueous extract revealed the presence of anthocyanins, flavonoids, tannins, phenolics, saponins, and reducing sugars, with a total phenolic content of 184.3 ± 5.2 mg GAE/g and a total flavonoid content of 92.7 ± 3.8 mg QE/g. Molecular docking showed that the identified compounds, such as linoleic acid methyl ester, exhibited strong binding affinities to bacterial DNA gyrase (up to −5.9 kcal/mol) and inflammatory targets, including COX‐2, 5‐LOX, and TNF‐α. ADMET predictions indicated favorable pharmacokinetic profiles for these compounds. These findings scientifically validate the traditional use of *H. sabdariffa* and highlight the potential of its aqueous extract as a promising source of bioactive compounds warranting further preclinical investigation for functional food and complementary health applications.

## Graphical abstract

This study presents a comprehensive evaluation of *Hibiscus sabdariffa* L. calyx extracts from Libya, integrating phytochemical profiling, multi‐target biological activities, and in silico validation. Aqueous and ethanolic extracts were prepared and assessed for antibacterial activity against multidrug‐resistant clinical isolates (MIC: 6.25–25 mg/mL; MBC/MIC ≤ 2), antioxidant capacity (IC_50_: 283.6–292.1 μg/mL), anti‐inflammatory effects via albumin denaturation inhibition (IC_50_: 129.81–379.46 μg/mL), and cytotoxicity using the brine shrimp lethality model (LD_50_: > 1000–345.5 μg/mL). GC–MS analysis of the ethanolic extract identified six major fatty acid methyl esters, with oleic acid methyl ester (41.01%) and palmitic acid methyl ester (28.76%) as the predominant compounds. The aqueous extract exhibited superior anti‐inflammatory activity (IC_50_: 129.81 μg/mL) and a non‐toxic profile (LD_50_ > 1000 μg/mL), while the ethanolic extract demonstrated potent bactericidal activity against *Staphylococcus aureus* (MIC: 6.25 mg/mL). Molecular docking revealed moderate binding affinities of the identified compounds to bacterial DNA gyrase (up to −5.9 kcal/mol) and anti‐inflammatory targets (COX‐2, 5‐LOX, TNF‐α; up to −7.7 kcal/mol). ADMET predictions indicated favorable pharmacokinetic profiles. These findings validate the traditional use of Libyan roselle and highlight its potential as a functional food candidate with multi‐target therapeutic applications.

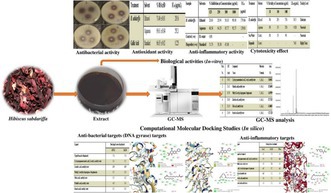

## 1 Introduction

Antimicrobial resistance (AMR) continues to escalate worldwide, posing severe challenges to public health systems. This trend compromises the effectiveness of existing antibiotics and underscores the pressing need for new therapeutic alternatives (Baker et al. 2022; Kabbashi et al. 2024; Organization 2023). In regions such as Libya and its neighboring countries, where healthcare challenges are compounded by resource limitations, the exploration of indigenous medicinal plants as complementary or alternative treatments is of paramount importance (Alzandi et al. 2023; Hosien et al. 2022).

*Hibiscus sabdariffa* L. has a long history of ethnomedicinal use. Its distinctive crimson calyces are not only popular for preparing refreshing drinks across several continents but also form the basis of traditional remedies aimed at alleviating a spectrum of ailments, from cardiovascular issues, such as hypertension, to metabolic disorders, fevers, and various infections (Duque‐Soto et al. 2023; Obu 2025). The reported health benefits of roselle are chemically underpinned by its diverse portfolio of secondary metabolites. This includes colored anthocyanins like delphinidin‐3‐sambubioside, a mixture of organic acids (e.g., hibiscus and citric acid), various flavonoid molecules, and complex carbohydrate polymers (Alizadeh et al. 2023; Gao et al. 2023). Consequently, scientific inquiries from different regions have consistently identified significant antibacterial, radical‐scavenging (antioxidant), and anti‐inflammatory properties in extracts derived from this plant (Harikrishnan and Balasundaram 2020; Zou et al. 2022). A critical consideration in phytomedicine is that a plant's chemical makeup, and thus its biological activity, is not fixed. It is dynamically shaped by the growth environment, including soil type, local weather patterns, and agricultural techniques (Palit and Mandal 2021). This implies that *H. sabdariffa* cultivated in a specific Libyan region, such as Al‐Bayda, could possess a unique chemical fingerprint, possibly leading to differentiated or even more potent biological effects than those of specimens from other locales (Phiri 2017).

Despite existing reports, a holistic comparative investigation of Libyan *H. sabdariffa* encompassing simultaneous phytochemical profiling, multi‐target biological evaluation (antibacterial, antioxidant, anti‐inflammatory, and cytotoxic), and in silico validation of the identified compounds remains scarce. Furthermore, while ethanolic extracts are commonly studied for their concentrated phytochemical content, aqueous extracts are more relevant to traditional preparation methods (e.g., infusions and decoctions) and may offer a more favorable safety profile (de Jesus et al. 2023; Lu et al. 2024).

This study was designed to bridge these gaps. We aimed to: (1) prepare and characterize ethanol and aqueous extracts from *H. sabdariffa* calyces (Al‐Bayda, Libya); (2) evaluate their in vitro antibacterial effects against multidrug‐resistant isolates, including MIC and MBC determination; (3) assess their antioxidant and anti‐inflammatory potentials; (4) determine their cytotoxicity using the brine shrimp lethality assay; (5) identify major phytoconstituents in the active extract via GC–MS; (6) perform preliminary phytochemical screening of the aqueous extract; and (7) perform in silico molecular docking (including free fatty acids and redocking validation) and ADMET (Absorption, Distribution, Metabolism, Excretion, and Toxicity) analyses of the identified compounds to predict their mechanisms of action and drug‐likeness. This integrated approach provides a scientific basis supporting the traditional use of Libyan roselle and highlights its potential as a source of multi‐functional bioactive compounds warranting further investigation for functional food development and for addressing AMR and inflammatory diseases.

## 2 Materials and Methods

### 2.1 Collection and Botanical Authentication of Plant Material

Fresh calyces of *Hibiscus sabdariffa* L. were harvested from plants cultivated in Al‐Bayda, Libya between May and July 2024. A voucher specimen (HS‐OMU‐2024‐025) was authenticated by a taxonomist and deposited in the Department of Botany, Faculty of Science, Omar Al‐Mukhtar University. Calyces were meticulously washed, shade‐dried at room temperature (25°C–30°C) for 2 weeks, ground into a fine powder using an electric grinder, and stored in airtight, light‐resistant containers at 4°C until extraction.

### 2.2 Preparation of Aqueous and Ethanolic Extracts

#### 2.2.1 Aqueous Extract

The dried powder, weighing 10 g, was mixed with 100 mL of distilled water (1:10 w/v). This mixture was stirred continuously using a magnetic stirrer at room temperature (25°C ± 2°C) for a period of 48 h. The mixture was filtered through a Whatman No. 1 filter paper. Afterward, the remaining solid material was soaked again in 50 mL of fresh solvent for 24 h. Thereafter, the mixed filtrates were concentrated using a rotary evaporator (Heidolph, Germany) at 40°C and reduced pressure. Different temperatures were used for rotary evaporation based on the boiling points of the solvents: water (boiling point 100°C) required a lower temperature (40°C) to prevent thermal degradation of heat‐labile anthocyanins and phenolic acids. The obtained semi‐solid extract was then dried in an oven at 35°C to create a dry powder.

#### 2.2.2 Ethanolic Extract

The dried powder (100 g) was macerated in 1 L of 75% ethanol (v/v, 1:10 w/v), subjected to continuous agitation for 3 days at ambient temperature (Harborne 1998). Subsequently, the mixture was filtered, and the ethanol was completely eliminated under reduced pressure at 55°C, employing a rotary evaporator. Ethanol (boiling point 78°C) allowed evaporation at 55°C without degradation. The concentrated extract was transferred to a pre‐weighed petri dish and allowed to dry completely at room temperature (25°C ± 2°C). The extraction yield (Y, %) was calculated using Equation (1):

(1) $$\text{Yield}\;\left(\%\right)=\frac{\text{Weight of the dried extract}\;\left(g\right)}{\text{Weight of the dried plant powder}\;\left(g\right)\;}\times 100$$

We stored the dried extracts at −20°C for subsequent analysis.

### 2.3 GC–MS Analysis

The ethanolic extract was selected for detailed characterization via Gas Chromatography–Mass Spectrometry (GC–MS) based on its superior yield of less polar compounds, which are particularly suitable for GC–MS analysis. The analysis was performed using an Agilent 7890 B GC system in conjunction with an Agilent 5977A MSD. Separation was accomplished using an HP‐5MS capillary column (30 m × 0.25 mm, 0.25 μm film thickness). The oven temperature was programmed to rise from 50 (maintained for 2 min) to 300°C at a rate of 10°C/min, followed by a final hold for 10 min. Helium was used as the carrier gas at a flow rate of 1 mL/min. The mass spectrometer operated in electron impact (EI) mode at 70 eV, with a scan range of m/z 50–550. Compound identification was performed by comparing the mass spectra with those in the NIST 17 library (match factor ≥ 850). Retention indices (RI) were calculated using a homologous series of n‐alkanes (C8–C40) and compared with literature values from the NIST Chemistry WebBook and PubChem databases. No derivatization was performed prior to analysis; the fatty acid methyl esters detected are naturally occurring constituents of *H. sabdariffa* calyces, consistent with previous reports (Çömlekcioğlu and Aygan 2020; Hounkpè et al. 2019). However, the possibility of minor transesterification during ethanolic extraction cannot be excluded.

### 2.4 Preliminary Phytochemical Screening

The dried aqueous extract was qualitatively tested for the presence of various phytoconstituents using standard protocols (Evans 2009; Sofowara 1993).

#### 2.4.1 Anthocyanins

Two milliliters of the extract were mixed with 2 mL of 2 M HCl and heated in a water bath for 5 min. The appearance of a pink‐red color indicated a positive result (Evans 2009).

#### 2.4.2 Flavonoids

Alkaline reagent test was performed by adding 2 mL of 1% NaOH to 2 mL of the extract. An intense yellow color that became colorless upon the addition of a few drops of dilute HCl indicated the presence of flavonoids (Sofowara 1993).

#### 2.4.3 Phenolics (General Test)

Two milliliter of extract was treated with 1 mL of 5% FeCl_3_ solution. A deep blue‐black or greenish‐black precipitate confirmed the presence of phenolic compounds (Evans 2009).

#### 2.4.4 Reducing Sugars

One milliliter of extract was mixed with 2 mL of Benedict's reagent and heated in a boiling water bath for 5 min. A brick‐red precipitate indicated the presence of reducing sugars (Sofowara 1993).

#### 2.4.5 Saponins

The froth test was conducted by adding 5 mL of distilled water to 2 mL of the extract and shaking vigorously for 30 s. The formation of a stable foam layer of more than 1 cm in height confirmed the presence of saponins (Evans 2009).

#### 2.4.6 Steroids

Two milliliter of extract was dissolved in 2 mL of chloroform, followed by the addition of 2 mL of concentrated H_2_SO_4_. A red color in the chloroform layer indicated a positive result, whereas no color change indicated a negative result (Sofowara 1993).

#### 2.4.7 Tannins

The extract (2 mL) was mixed with 2 mL of 5% FeCl_3_ solution. The presence of tannins was confirmed by the formation of a blue‐black or greenish‐black precipitate; confirmed the presence of tannins (Evans 2009).

#### 2.4.8 Terpenoids

The Salkowski test was performed by mixing 2 mL of the extract with 2 mL of chloroform and 2 mL of concentrated H_2_SO_4_. A reddish‐brown interface indicated a positive result, while the absence of this color indicated a negative result (Sofowara 1993).

### 2.5 Quantification of Total Phenolic and Flavonoid Contents

#### 2.5.1 Determination of Total Phenolic Content (TPC)

The total phenolic content was determined using the Folin–Ciocalteu method (Singleton et al. 1999). Briefly, 0.5 mL of the aqueous extract (1 mg/mL) was mixed with 2.5 mL of 10% Folin–Ciocalteu reagent and allowed to stand for 5 min. Then, 2 mL of 7.5% Na_2_CO_3_ solution was added, and the mixture was incubated in the dark at room temperature for 30 min. The absorbance was measured at 765 nm using UV–Vis spectrophotometry. Gallic acid was used as the standard, and the results were expressed as mg gallic acid equivalents per gram of extract (mg GAE/g) of the extract. All measurements were performed in triplicates.

#### 2.5.2 Determination of Total Flavonoid Content (TFC)

The total flavonoid content was determined using the aluminum chloride colorimetric method (Zhishen et al. 1999). One milliliter of the extract (1 mg/mL) was mixed with 3 mL of methanol, 0.2 mL of 10% AlCl_3_, 0.2 mL of 1 M potassium acetate, and 5.6 mL of distilled water. The mixture was incubated at room temperature for 30 min, and the absorbance was measured at 510 nm. Quercetin was used as the standard, and the results were expressed as mg quercetin equivalents per gram of extract (mg QE/g extract). All measurements were performed in triplicates.

### 2.6 Antibacterial Susceptibility Testing

#### 2.6.1 Bacterial Isolates

Clinical bacterial isolates, including *Staphylococcus aureus* , *Pseudomonas aeruginosa* , *Escherichia coli* , *Klebsiella pneumoniae* , and *Proteus vulgaris* , were obtained from the diagnostic laboratories at the Al‐Bayda Medical Center. Isolates were selected based on their clinical prevalence in Libya and documented multidrug‐resistant profiles (CLSI 2018). All isolates were anonymized and used under institutional Approval No Omar Al‐Mukhtar University Research Ethics Committee (Approval No. OMU‐REC‐2024‐089, dated March 15, 2024). Prior to testing, the isolates were sub‐cultured on Mueller‐Hinton Agar (MHA) to ensure purity and viability.

#### 2.6.2 Agar Well Diffusion Assay

The antibacterial effectiveness of the extracts was assessed using the agar well diffusion method, adhering to a standardized methodology with minor modifications (CLSI 2018). Bacterial suspensions were calibrated to a turbidity of 0.5 McFarland standard, corresponding to approximately 1.5 × 10^8^ CFU/mL in sterile saline solution. Mueller–Hinton Agar plates were evenly inoculated with bacterial suspensions. Aseptically, wells (6 mm in diameter) were created in the agar plates. Each well was filled with 100 μL of the extract solution (100 mg/mL). For comparison, standard antibiotics (augmentin, ciprofloxacin, and azithromycin) were tested using the same well diffusion method at a concentration of 50 μg/mL. Control wells containing 10% DMSO and sterile distilled water were included on the same plate. The plates were subsequently incubated at 37°C for 18–24 h. The inhibition zone diameters (IZD) were measured in millimeters (mm). All assays were conducted in triplicate using three independent extract batches (biological replicates).

#### 2.6.3 Determination of Minimum Inhibitory Concentration (MIC) and Minimum Bactericidal Concentration (MBC)

The MIC of both extracts was determined using the broth microdilution method, according to CLSI guidelines (M07‐A11). Two‐fold serial dilutions of each extract (0.39–200 mg/mL) were prepared in Mueller‐Hinton Broth (MHB) in 96‐well plates. Bacterial suspensions were adjusted to the 0.5 McFarland standard (approximately 1.5 × 10^8^ CFU/mL) and further diluted to approximately 5 × 10^5^ CFU/mL. After 18–24 h of incubation at 37°C, the MIC was recorded as the lowest concentration showing no visible growth. The MBC was determined by subculturing 10 μL from wells showing no growth onto Mueller‐Hinton Agar; the MBC was the lowest concentration killing ≥ 99.9% of the initial inoculum. The MBC/MIC ratio was calculated to determine bactericidal (≤ 4) and bacteriostatic (> 4) activities.

### 2.7 Antioxidant Activity (DPPH Radical Scavenging Assay)

The free radical scavenging ability, which reflects the antioxidant capability, was measured using the DPPH test (Plank et al. 2012). This approach relies on the conversion of the purple‐hued DPPH radical into a yellow‐hued substance in the presence of an antioxidant. Serial dilutions of the extracts were combined with a methanolic DPPH solution, and the reduction in absorbance at 517 nm was recorded after a specified incubation period in the dark. The percentage radical scavenging activity (% RSA) was determined using Equation (2):

(2) $$\left(\%\right)\;\mathrm{RSA}=\left\{\frac{A_{\text{control}}-A_{\text{sample}}\;}{A_{\text{control}}}\right\}\times 100$$

To calculate the IC_50_ (concentration for 50% radical scavenging), % RSA was plotted against concentration, and the data were analyzed by non‐linear regression using GraphPad Prism 9.0.

### 2.8 In Vitro Anti‐Inflammatory Activity (Albumin Denaturation Inhibition)

The anti‐inflammatory properties were assessed using an in vitro model based on the inhibition of heat‐induced denaturation of egg albumin protein (Ranaweera et al. 2023). For the assessment, varying concentrations of the crude extracts (125–1000 μg/mL) and the reference drug ibuprofen (125–500 μg/mL) were formulated using distilled water. For the protein denaturation inhibition assay, 0.2 mL of fresh egg albumin was mixed with 2.8 mL of PBS (pH 6.4) and 2 mL of the sample solution. This mixture was first maintained at 37°C for 15 min and then heated to 70°C for an additional 5 min in a water bath. After allowing the samples to cool to ambient temperature, their turbidity was quantified by measuring the absorbance at 660 nm. A control sample was prepared in parallel by substituting the test solution with an equal volume of distilled water for comparison. The extent of protein denaturation inhibition was subsequently determined using the following Equation (3):

(3) $$\text{Inhibition}\;\left(\%\right)=\left\{\frac{A_{\text{control}}-A_{\text{sample}}\;}{A_{\text{control}}}\right\}\times 100$$

The IC_50_ value was calculated from the dose–response curve.

### 2.9 Cytotoxicity Assessment (Brine Shrimp Lethality Assay)

A preliminary evaluation of cytotoxicity was performed with the brine shrimp ( *Artemia salina* ) lethality test, a common preliminary screening tool (McLaughlin et al. 1998; Meyer et al. 1982). The brine shrimp lethality assay involved the invertebrate species *Artemia salina* ; therefore, formal animal ethics approval was not required under Libyan national regulations. Nevertheless, all procedures were conducted in accordance with institutional guidelines for the humane treatment of live organisms. *Artemia salina* eggs were incubated in synthetic seawater (created by dissolving 38 g/L of marine salt in distilled water) with continuous oxygen supply and light exposure. The hatching process lasted 48 h within a temperature range of 25°C–28°C. Ten actively swimming nauplii (larval stage) were transferred to vials containing 5 mL of seawater and different extract concentrations (10, 100, and 1000 μg/mL). Each concentration was tested in triplicates. A negative control (seawater only) and positive control (etoposide, 10 μg/mL) were used. The vials were incubated for 24 h under illumination. The number of dead nauplii in each vial was then counted. The percentage mortality was calculated, and the median lethal concentration (LD_50_) was determined via probit analysis using SPSS software (v26). Based on the median lethal concentration (LD_50_), toxicity was categorized using established criteria: highly toxic (LD_50_ < 250 μg/mL), moderately toxic (250–499 μg/mL), slightly toxic (500–1000 μg/mL), and non‐toxic (LD_50_ > 1000 μg/mL) (McLaughlin et al. 1998).

### 2.10 Computational Molecular Docking Studies

To investigate the possible modes of action of the key phytoconstituents, computational molecular docking simulations were conducted. The three‐dimensional atomic coordinates of the relevant protein targets were obtained from the Protein Data Bank (PDB). To explore the antibacterial mechanisms, DNA gyrase subunit B from *Staphylococcus aureus* (PDB: 6TTG) and *Escherichia coli* (PDB: 1KZN) were selected. To examine the anti‐inflammatory potential, targets included Cyclooxygenase‐2 (COX‐2, PDB: 6COX), 5‐Lipoxygenase (5‐LOX, PDB: 1CYF), and Tumor Necrosis Factor‐alpha (TNF‐α, PDB: 3F4M). These protein structures were prepared for docking by eliminating water molecules, incorporating polar hydrogen atoms, and applying Kollman partial atomic charges using the AutoDock Tools (v1.5.6) software package (version 1.5.6).

For proteins with missing residues in the crystal structure (e.g., 1CYF with missing loop regions), the existing residues were retained without modeling the missing segments, as these regions were not within the defined binding pocket. The docking grid was specifically centered on the known active site (for 1CYF, centered on the iron‐binding site defined by His367, His372, and Ile406), minimizing the impact of the missing peripheral residues.

1CYF (5‐lipoxygenase) is a crystallized construct containing surface mutations (E356Q, K361E, K362Q, K363E, K364Q, K365E, and K366Q) designed to improve crystallization and stability. This is the only high‐resolution 5‐LOX structure available (2.35 Å resolution) and is widely used in docking studies. The mutations are located on the surface of the protein, distant from the active site, and are therefore not expected to significantly affect the ligand‐binding affinity. Alternative structures (e.g., 3O8Y) have lower resolution or are complexed with inhibitors that alter the binding pocket conformation.

The two‐dimensional structures of the six principal chemical compounds identified using GC–MS (cyclopropaneoctanoic acid, 2‐octyl‐, methyl ester; linoleic acid, methyl ester; methyl 2‐octylcyclopropene‐1‐heptanoate; oleic acid, methyl ester; palmitic acid, methyl ester; and stearic acid, methyl ester) and their corresponding free fatty acids and reference pharmaceuticals (Ciprofloxacin, Ibuprofen) were illustrated in ChemDraw and subsequently converted to three‐dimensional models and energy‐minimized using the MM2 force field in Avogadro software.

Docking simulations were conducted using AutoDock Vina (v1.2.0), with the search space delineated to include the active site of each protein. Grid box dimensions were as follows: 6TTG (center x = 6.995, y = 7.171, z = −3.471; size 25 × 25 × 25 Å); 1KZN (center x = 20.154, y = 23.677, z = 35.508; size 25 × 25 × 25 Å); 6COX (center x = 35.757, y = 11.979, z = 29.521; size 30 × 30 × 30 Å); 1CYF (center x = 38.264, y = −30.849, z = 8.178; size 25 × 25 × 25 Å); 3F4M (center x = 64.580, y = 9.418, z = 10.476; size 26 × 26 × 26 Å). The exhaustiveness was set to 16.

For validation, the co‐crystallized ligands were re‐docked into their respective binding sites, and the RMSD between the docked pose and crystal structure conformation was calculated. RMSD values ≤ 2.0 Å were considered acceptable for validation. Redocking of ciprofloxacin into 6TTG yielded an RMSD of 1.4 Å, and redocking of ibuprofen into 6COX yielded an RMSD of 1.2 Å, confirming the accuracy of the docking protocol.

While free fatty acids are generally considered biologically active, methyl esters were docked as they represent the compounds identified by GC–MS and are stable during analysis. Additionally, methyl esters can be hydrolyzed to free fatty acids in vivo by esterases, and docking both forms provides a more complete picture of their potential bioactivity.

The optimal conformation was identified based on the binding affinity (kcal/mol). Protein‐ligand interactions were depicted using PyMOL (v2.5) and Discovery Studio Visualizer (V25.1.0).

### 2.11 ADMET Property Prediction

The ADMET profiles of the six identified chemicals were predicted using the online servers ADMETLab 2.0 (https://admetmesh.scbdd.com/) and SwissADME (http://www.swissadme.ch/). The predicted parameters included human intestinal absorption (HIA), Caco‐2 permeability, blood–brain barrier (BBB) penetration, plasma protein binding (PPB), cytochrome P450 inhibition, clearance, half‐life, hERG inhibition, and oral rat acute toxicity (LD_50_). Drug‐likeness was assessed using Lipinski, Ghose, Veber, Egan, and Pfizer rules.

### 2.12 Statistical Analysis

All laboratory experiments were performed in triplicate using three independently prepared extract batches (biological replicates, *n* = 3) from the same pooled plant material. For each biological replicate, assays were conducted in triplicate wells (technical replicates). Data are presented as mean ± standard deviation (SD) of the three biological replicates. One‐way analysis of variance (ANOVA) followed by Tukey's post hoc test was used for comparisons involving multiple groups (e.g., inhibition zone diameters across bacterial strains). Unpaired two‐tailed Student's *t*‐test was used for comparisons between two groups (e.g., aqueous vs. ethanolic extract IC_50_ values). Exact *p*‐values are reported where statistically significant differences were observed (*p* < 0.05). All analyses were performed using SPSS version 26 and GraphPad Prism version 10.0.

## 3 Results

### 3.1 Extraction Yield

The extraction yields and physical characteristics are listed in Table 1. The aqueous extract yielded a significantly higher percentage (51.10% ± 2.15%) compared to the ethanolic extract (33.48% ± 1.82%). This difference is consistent with the high water solubility of polar constituents such as polysaccharides, organic acids (e.g., hibiscus and citric acids), and glycosylated anthocyanins. Both extracts exhibited a deep red color, characteristic of *H. sabdariffa* anthocyanins, and a gummy consistency.

**TABLE 1:** Yield and physical characteristics of *H. sabdariffa* calyx extracts.

Scientific name	Family	Solvent used	Yield (%)	Color	Consistency
*H. sabdariffa*	Malvaceae	Ethanol (75%)	33.48 ± 1.82	Deep red	Gummy
*H. sabdariffa*	Malvaceae	Aqueous	51.10 ± 2.15	Deep red	Gummy

*Note:* Values are presented as mean ± SD (*n* = 3 independent extract batches).

### 3.2 Phytochemical Profiling

#### 3.2.1 GC–MS Analysis of Ethanolic Extract

**FIGURE 1:**
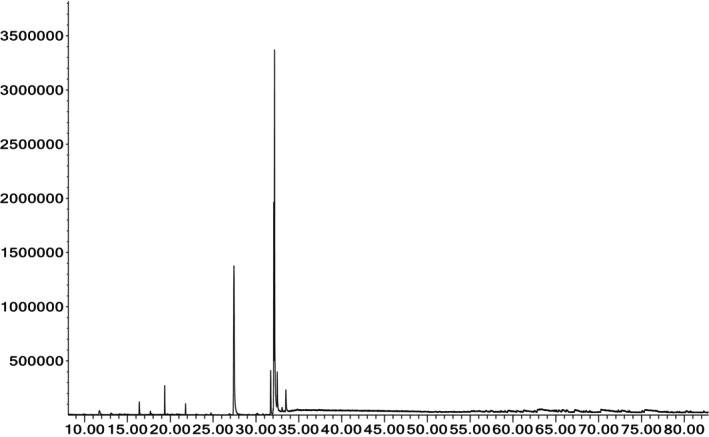
Representative GC–MS chromatogram of the ethanol extract of *H. sabdariffa* calyces.

GC–MS analysis of the ethanolic extract identified six major compounds, representing over 95% of the total chromatographic area (Table 2 and Figure 1). The predominant compound was oleic acid methyl ester (41.01%), followed by palmitic acid methyl ester (28.76%) and linoleic acid methyl ester (17.57%). Other significant constituents included stearic acid methyl ester (5.27%), methyl 2‐octylcyclopropene‐1‐heptanoate (4.37%), and cyclopropaneoctanoic acid 2‐octyl‐, methyl ester (3.02%). The prevalence of fatty acid methyl esters was consistent with previous reports on *H. sabdariffa* seed oils and calyx extracts.

**TABLE 2:** Phytoconstituents identified in the ethanolic extract of *H. sabdariffa* by GC–MS.

Peak no.	RT (min)	Compound name	Molecular formula	Area (%)	Calculated RI	Literature RI
1	33.49	Cyclopropaneoctanoic acid, 2‐octyl‐, methyl ester	C_20_H_38_O_2_	3.02	2156	2148
2	27.43	Palmitic acid, methyl ester	C_17_H_34_O_2_	28.76	1924	1922
3	31.72	Methyl 2‐octylcyclopropene‐1‐heptanoate	C_19_H_34_O_2_	4.37	2089	2083
4	32.09	Linoleic acid, methyl ester	C_19_H_34_O_2_	17.57	2105	2100
5	32.17	Oleic acid, methyl ester	C_19_H_36_O_2_	41.01	2108	2104
6	32.49	Stearic acid, methyl ester	C_19_H_38_O_2_	5.27	2119	2116

#### 3.2.2 Preliminary Phytochemical Screening of Aqueous Extract

The aqueous extract was not amenable to GC–MS analysis because of the non‐volatile, polar nature of its constituents. Preliminary phytochemical screening using standard qualitative tests revealed the presence of anthocyanins, flavonoids, tannins, phenolics, saponins, and reducing sugars, while terpenoids and steroids were absent (Table 3). Quantitative analysis revealed a total phenolic content of 184.3 ± 5.2 mg GAE/g extract and a total flavonoid content of 92.7 ± 3.8 mg QE/g extract.

**TABLE 3:** Preliminary phytochemical screening and quantitative analysis of the aqueous extract.

Compound	Qualitative result	Quantitative content	Unit
Anthocyanins	Positive (+)	—	—
Flavonoids	Positive (+)	92.70 ± 3.80	mg QE/g
Phenolics	Positive (+)	184.30 ± 5.21	mg GAE/g
Reducing Sugars	Positive (+)	—	—
Saponins	Positive (+)	—	—
Steroids	Negative (−)	—	—
Tannins	Positive (+)	—	—
Terpenoids	Negative (−)	—	—

*Note:* (+): indicates the presence of the compound; (−): indicates the absence of the compound. Values are expressed as the mean ± SD (*n* = 3).

Abbreviations: GAE, Gallic acid equivalent; QE, Quercetin equivalent.

### 3.3 Antibacterial Activity

#### 3.3.1 Agar Well Diffusion Assay

**FIGURE 2:**
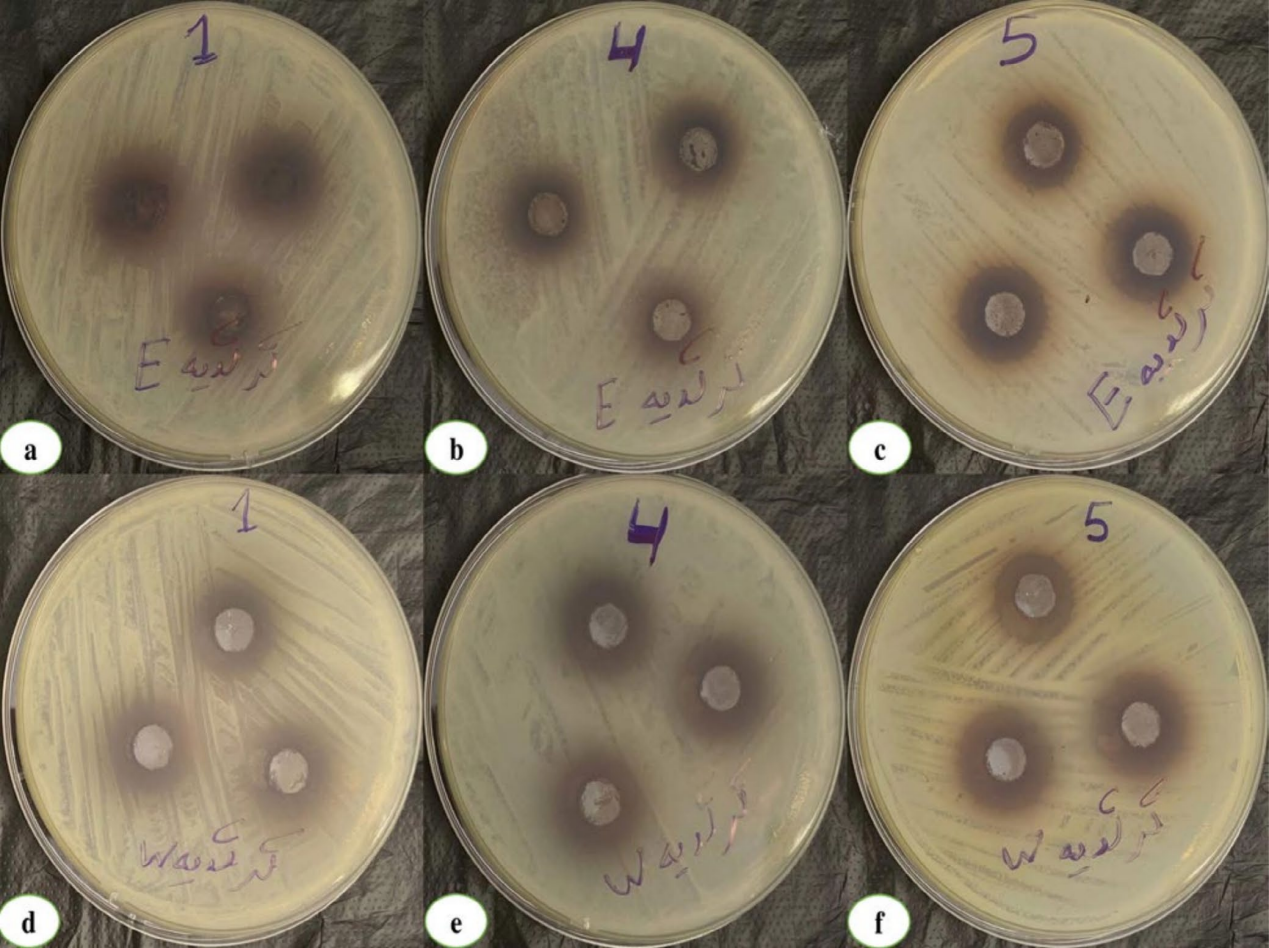
Antibacterial activity of *H. sabdariffa* extracts (100 mg/mL) against clinical bacterial isolates. (a–c) Ethanolic extract: (a) *S. aureus* , (b) *K. pneumoniae* , (c) *P. vulgaris* . (d–f) Aqueous extract: (d) *S. aureus* , (e) *K. pneumoniae* , (f) *P. vulgaris* .

Both extracts exhibited broad‐spectrum antibacterial activity against the tested clinical isolates (Table 4 and Figure 2). The activity was solvent‐dependent, reflecting the differential extraction of the bioactive compounds.

**TABLE 4:** Antibacterial activity of *H. sabdariffa* extracts by agar well diffusion method.

Treatment	Solvent/type	*S. aureus*	*E. coli*	*P. aeruginosa*	*K. pneumoniae*	*P. vulgaris*
		Mean inhibition zone diameter (mm) ± SD				
*H. sabdariffa*	Ethanol	20.0 ± 1.00^a^	15.0 ± 0.00^b^	17.6 ± 0.50^b^	14.3 ± 0.50^b^	18.0 ± 1.00^ab^
	Aqueous	17.6 ± 0.50^b^	13.6 ± 0.50^c^	20.0 ± 1.73^a^	18.3 ± 1.52^a^	19.0 ± 0.00^a^
DMSO 10%	Negative control Negative control	0.00^d^	0.00^e^	0.00^d^	0.00^d^	0.00^d^
Distilled Water		0.00^d^	0.00^e^	0.00^d^	0.00^d^	0.00^d^
Azithromycin	Positive control Positive control Positive control	16.3 ± 0.40^c^	0.00^e^	0.00^d^	0.00^d^	0.00^d^
Augmentin		20.0 ± 0.00^a^	20.3 ± 0.57^a^	16.6 ± 0.52^c^	0.00^d^	0.00^d^
Ciprofloxacin		24.0 ± 0.58^a^	26.3 ± 0.82^a^	21.0 ± 0.82^c^	20.5 ± 0.58^a^	19.5 ± 0.58^ab^

*Note:* Values are expressed as mean ± SD (*n* = 3 biological replicates). Superscript letters (a–e) within each column indicate significant differences by Tukey's HSD test (*p* < 0.05). Exact *p*‐values for pairwise comparisons are provided in the text (Section 3.3.1). Verification: The exact *p*‐values (*p* = 0.031, *p* = 0.045, *p* = 0.008) were already present in Sections 3.3.1 and 3.5 and remain unchanged.

Abbreviations: DMSO, dimethyl sulfoxide; SD, standard deviation.

The ethanolic extract exhibited potent activity against Gram‐positive *S. aureus* (20.0 ± 1.00 mm) and Gram‐negative *P. vulgaris* (18.0 ± 1.00 mm). The ethanolic extract was significantly more active against *S. aureus* than the aqueous extract (20.0 ± 1.00 mm vs. 17.6 ± 0.50 mm, *p* = 0.031, unpaired *t*‐test).

The aqueous extract demonstrated remarkable efficacy against challenging Gram‐negative pathogens: *P. aeruginosa* (20.0 ± 1.73), *K. pneumoniae* (18.3 ± 1.52), and *P. vulgaris* (19.0 ± 0.00 mm). Notably, the aqueous extract was significantly more effective against *P. aeruginosa* than the ethanolic extract (20.0 ± 1.73 mm vs. 17.6 ± 0.50 mm, *p* = 0.045, unpaired *t*‐test). Importantly, isolates resistant to conventional antibiotics (e.g., *K. pneumoniae* resistant to azithromycin and Augmentin and *P. vulgaris* resistant to Augmentin) remained susceptible to both extracts.

#### 3.3.2 Minimum Inhibitory and Bactericidal Concentrations

The MIC values ranged from 6.25 to 25 mg/mL, with corresponding MBC values ranging from 12.5 to 50 mg/mL (Table 5). The MBC/MIC ratios were ≤ 2 for all extract‐bacteria combinations, confirming bactericidal activity. Notably, the aqueous extract demonstrated the lowest MIC (6.25 mg/mL) against *P. aeruginosa* and *P. vulgaris* , whereas the ethanolic extract was most effective against *S. aureus* (MIC: 6.25 mg/mL).

**TABLE 5:** Minimum inhibitory concentration (MIC) and minimum bactericidal concentration (MBC) of *H. sabdariffa* extracts (mg/mL).

Bacterial strain	Extract	MIC (mg/mL)	MBC (mg/mL)	MBC/MIC ratio	Activity type
*S. aureus*	Ethanol	6.25	12.5	2	Bactericidal
*S. aureus*	Aqueous	12.5	25.0	2	Bactericidal
*E. coli*	Ethanol	25.0	50.0	2	Bactericidal
*E. coli*	Aqueous	50.0	100.0	2	Bactericidal
*P. aeruginosa*	Ethanol	12.5	25.0	2	Bactericidal
*P. aeruginosa*	Aqueous	6.25	12.5	2	Bactericidal
*K. pneumoniae*	Ethanol	25.0	50.0	2	Bactericidal
*K. pneumoniae*	Aqueous	12.5	25.0	2	Bactericidal
*P. vulgaris*	Ethanol	12.5	12.5	1	Bactericidal
*P. vulgaris*	Aqueous	6.25	12.5	2	Bactericidal

### 3.4 Antioxidant Activity

Both extracts exhibited strong and statistically comparable DPPH radical‐scavenging capacities (Table 6). The IC_50_ values were 283.6 and 292.1 μg/mL for the ethanolic and aqueous extracts, respectively. The difference was not statistically significant (*p* = 0.382, unpaired *t*‐test), indicating that both polar and semi‐polar solvents effectively extracted antioxidant compounds.

**TABLE 6:** Antioxidant activity of *H. sabdariffa* extracts by DPPH assay.

Treatment	Solvent/type	% RSA ± SD	IC_50_ (μg/mL)
*H. sabdariffa*	Ethanol	71.40 ± 0.01	283.6
*H. sabdariffa*	Aqueous	69.61 ± 0.04	292.1
Gallic Acid	Positive control	66.83 ± 0.02	1.225

*Note:* Values are expressed as mean ± SD (*n* = 3).

Abbreviation: RSA, radical scavenging activity.

The IC_50_ values of the extracts were substantially higher than that of the standard gallic acid (1.225 μg/mL). This difference is expected because (1) gallic acid is a pure compound optimized for radical scavenging, whereas the extracts are complex mixtures; (2) the extracts contain non‐antioxidant components that dilute the effective concentration of bioactive phenolics; and (3) synergistic or antagonistic interactions among multiple compounds may affect the overall activity.

### 3.5 Anti‐Inflammatory Activity

The anti‐inflammatory activity, assessed by the inhibition of albumin denaturation, revealed marked differences between the extracts (Table 7). The aqueous extract exhibited superior concentration‐dependent inhibition, with an IC_50_ of 129.81 μg/mL, approximately three times lower (indicating higher potency) than that of the ethanolic extract (IC_50_: 379.46 μg/mL). This difference was statistically significant (*p* = 0.008, one‐way ANOVA, Tukey's post hoc test).

**TABLE 7:** Anti‐inflammatory activity of *H. sabdariffa* extracts by protein denaturation assay.

Sample	Solvent/type	% inhibition at concentration (μg/mL)				IC_50_ (μg/ml)
		125	250	500	1000	
*H. sabdariffa*	Ethanol	20.80 ± 0.04	20.94 ± 0.09	56.03 ± 0.01	90.98 ± 0.05	379.46
*H. sabdariffa*	Aqueous	48.54 ± 0.08	64.35 ± 0.02	83.77 ± 0.03	92.37 ± 0.01	129.81
Distilled water	Negative control	0.00 ± 0.00	0.00 ± 0.00	0.00 ± 0.00	0.00 ± 0.00	—
Ibuprofen (+ve)	Positive control	31.70 ± 0.01	38.30 ± 0.03	43.88 ± 0.07	—	—

*Note:* Values are mean ± SD (*n* = 3).

At lower concentrations, the aqueous extract showed greater percentage inhibition compared to ibuprofen (e.g., 48.54% ± 0.08% at 125 μg/mL vs. 31.70% ± 0.01% for ibuprofen). However, these results should be interpreted cautiously, as the albumin denaturation assay is an in vitro surrogate model and does not directly measure inhibition of cyclooxygenase‐2 (COX‐2), 5‐lipoxygenase (5‐LOX), or pro‐inflammatory cytokine production. Confirmatory studies using enzymatic and cellular assays are required.

### 3.6 Cytotoxicity (Brine Shrimp Lethality Assay)

Cytotoxicity assessment revealed a critical distinction between the safety profiles of these two extracts (Table 8). The aqueous extract showed no significant lethality at the concentrations tested, with an LD_50_ > 1000 μg/mL. In contrast, the ethanolic extract exhibited moderate toxicity, with an LD_50_ of 345.5 μg/mL. The brine shrimp lethality assay serves as a preliminary screening tool; however, these results should be confirmed using validated mammalian cell lines (e.g., MTT assay on Vero, HepG2, or RAW 264.7 cells) before therapeutic or food applications.

**TABLE 8:** Cytotoxicity of *H. sabdariffa* extracts by brine shrimp lethality assay.

Treatment	Solvent/type	% mortality at concentration (μg/mL)			LD_50_ (μg/mL)	Toxicity level
		10	100	1000		
*H. sabdariffa*	Ethanol	0%	20%	70%	345.5	Moderate
*H. sabdariffa*	Aqueous	0%	0%	30%	> 1000	Non‐toxic
Sea Water	Negative control	0%	0%	0%	—	Non‐toxic
Etoposide	Positive control	—	—	—	7.463	Highly Toxic

*Note:* Toxicity Classification: LD_50_ < 250: highly toxic; 250–499: moderately toxic; 500–1000: lightly toxic; > 1000 μg/mL: non‐toxic (McLaughlin et al. 1998).

### 3.7 Computational Molecular Docking Studies

Molecular docking was performed to investigate the potential mechanisms of action underlying the observed antibacterial and anti‐inflammatory activities. The six major compounds identified by GC–MS (Table 2) were docked into five protein targets: bacterial DNA gyrase from *S. aureus* (6TTG) and *E. coli* (1KZN), COX‐2 (6COX), 5‐LOX (1CYF), and TNF‐α (3F4M).

All six compounds showed moderate binding affinities to bacterial DNA gyrase, with values ranging from −4.9 to −5.9 for methyl esters and −4.9 to −6.0 kcal/mol for free fatty acids (Table 9 and Table S1). Against anti‐inflammatory targets, the binding energies ranged from −5.4 to −7.7 for methyl esters and −5.9 to −8.1 kcal/mol for free fatty acids, with cyclopropaneoctanoic acid methyl ester showing the strongest binding to 5‐LOX (−7.7 kcal/mol) (Table 10 and Table S2).

**TABLE 9:** Molecular docking scores of methyl esters against bacterial DNA gyrase (kcal/mol).

Ligand	Docking scores (kcal/mol)		Key hydrogen bonds
	6TTG ( *S. aureus* )	1KZN ( *E. coli* )	
Ciprofloxacin (Standard drug)	−7.8	−7.8	**6TTG:** GLY85, THR173; **1KZN:** ARG76, ARG136, THR165.
Cyclopropaneoctanoic acid, 2‐octyl‐, methyl ester	−5.4	−5.7	**1KZN:** ASN46, VAL120, SER121.
Linoleic acid, methyl ester	**−5.9**	**−5.9**	**1KZN:** ASN46, VAL120, SER121.
Methyl 2‐octylcyclopropene‐1‐heptanoate	−5.7	−5.5	**6TTG:** ASN54, SER55.
Oleic acid, methyl ester	−5.5	−5.2	**6TTG:** ASN54; **1KZN:** ASN46, ASN120, SER121.
Palmitic acid, methyl ester	−4.9	−5.4	**6TTG:** SER55; **1KZN:** ASN46, VAL120.
Stearic acid, methyl ester	−5.3	−5.1	**6TTG:** ARG81; **1KZN:** ASN46.

**TABLE 10:** Molecular docking scores of methyl esters against anti‐inflammatory targets (kcal/mol).

Ligand	Docking scores (kcal/mol)			Key hydrogen bonds
	COX‐2 (6COX)	5‐LOX (1CYF)	TNF‐α (3F4M)	
Ibuprofen (standard drug)	−6.7	−7.9	−6.5	**Cox‐2:** ARG469, GLU465; **5‐LOX:** LEU177; **TNF‐α:** ALA67.
Cyclopropaneoctanoic acid, 2‐octyl‐, methyl ester	−5.9	**−7.7**	**−6.0**	**Cox‐2:** ASN43, ALA156; **5‐LOX:** LEU177.
Linoleic acid, methyl ester	−5.9	**−7.6**	−5.9	**Cox‐2:** ASN43; **5‐LOX:** VAL45.
Methyl 2‐octylcyclopropene‐1‐heptanoate	**−6.7**	**−7.7**	**−6.1**	**Cox‐2:** ARG44; **5‐LOX:** THR234.
Oleic acid, methyl ester	**−6.3**	−7.2	−5.5	**Cox‐2:** ASN43; **5‐LOX:** ARG48.
Palmitic acid, methyl ester	−5.6	−7.2	−5.4	**Cox‐2:** ASN43.
Stearic acid, methyl ester	−5.8	−7.2	−5.4	**Cox‐2:** ARG44.

Redocking validation yielded RMSD values ≤ 1.9 Å for all targets (Table S3). Detailed binding interactions 2D and 3D poses are provided in Figures 3, 4, 5, 6, 7 and Figures S1–S5 for methyl esters and Figures S6–S15 for free fatty acids.

**FIGURE 3:**
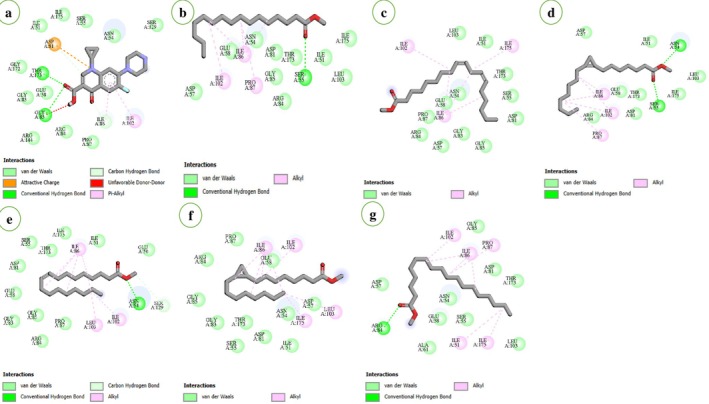
2D binding interactions of DNA gyrase (6TTG) with (a) Ciprofloxacin (standard drug); (b) Cyclopropaneoctanoic acid, 2‐octyl‐, methyl ester; (c) Linoleic acid, methyl ester; (d) Methyl 2‐octylcyclopropene‐1‐heptanoate; (e) Oleic acid, methyl ester; (f) Palmitic acid, methyl ester; (g) Stearic acid, methyl ester.

**FIGURE 4:**
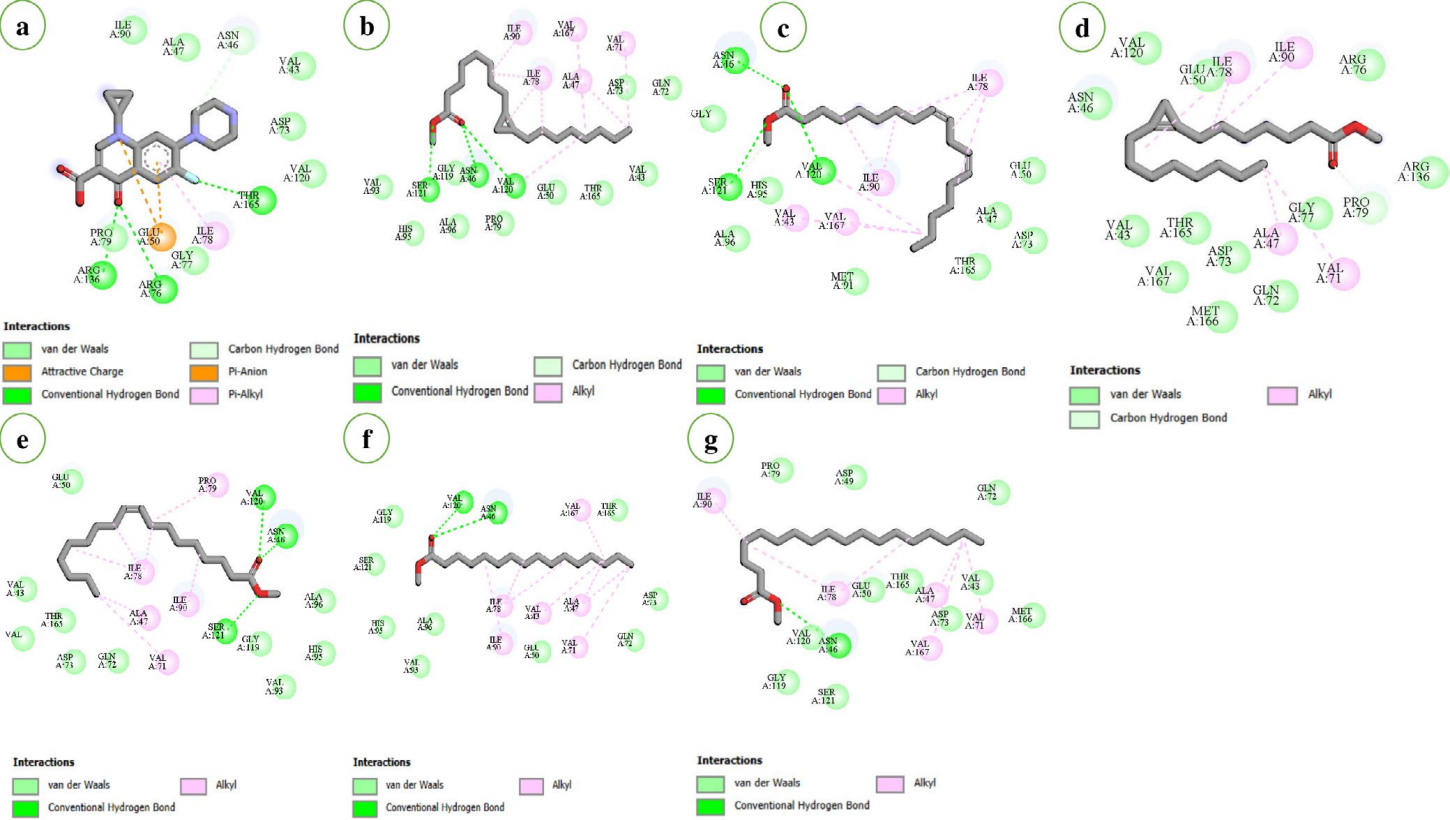
2D binding interactions of DNA gyrase (1KZN) with (a) Ciprofloxacin (standard drug); (b) Cyclopropaneoctanoic acid, 2‐octyl‐, methyl ester; (c) Linoleic acid, methyl ester; (d) Methyl 2‐octylcyclopropene‐1‐heptanoate; (e) Oleic acid, methyl ester; (f) Palmitic acid, methyl ester; (g) Stearic acid, methyl ester.

**FIGURE 5:**
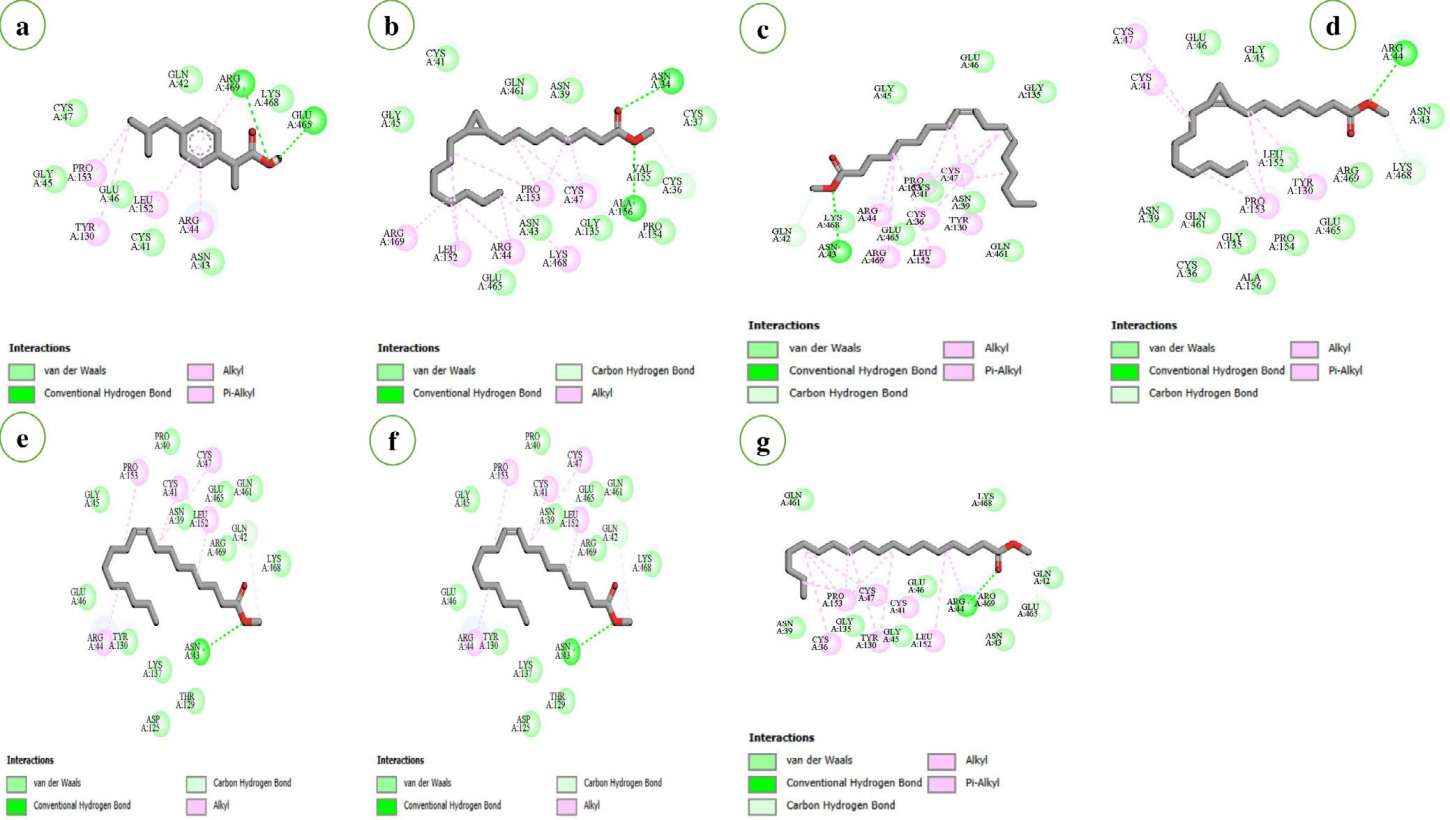
2D binding interactions of COX‐2 with (a) Ibuprofen (standard drug); (b) Cyclopropaneoctanoic acid, 2‐octyl‐, methyl ester; (c) Linoleic acid, methyl ester; (d) Methyl 2‐octylcyclopropene‐1‐heptanoate; (e) Oleic acid, methyl ester; (f) Palmitic acid, methyl ester; (g) Stearic acid, methyl ester.

**FIGURE 6:**
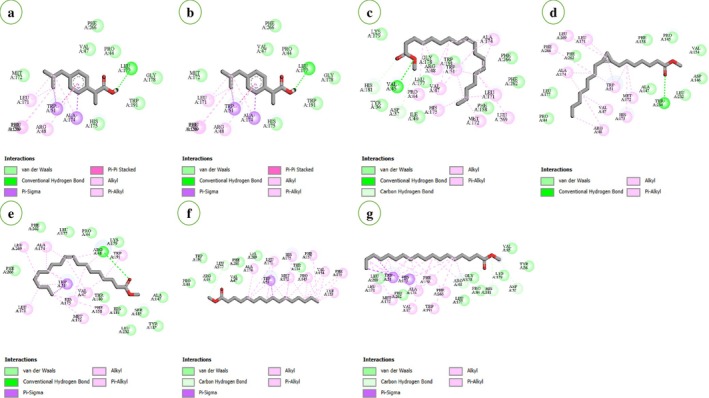
2D binding interactions of 5‐LOX with (a) Ibuprofen (standard drug); (b) cyclopropaneoctanoic acid, 2‐octyl‐, methyl ester; (c) linoleic acid, methyl ester; (d) methyl 2‐octylcyclopropene‐1‐heptanoate; (e) oleic acid, methyl ester; (f) palmitic acid, methyl ester; and (g) stearic acid, methyl ester.

**FIGURE 7:**
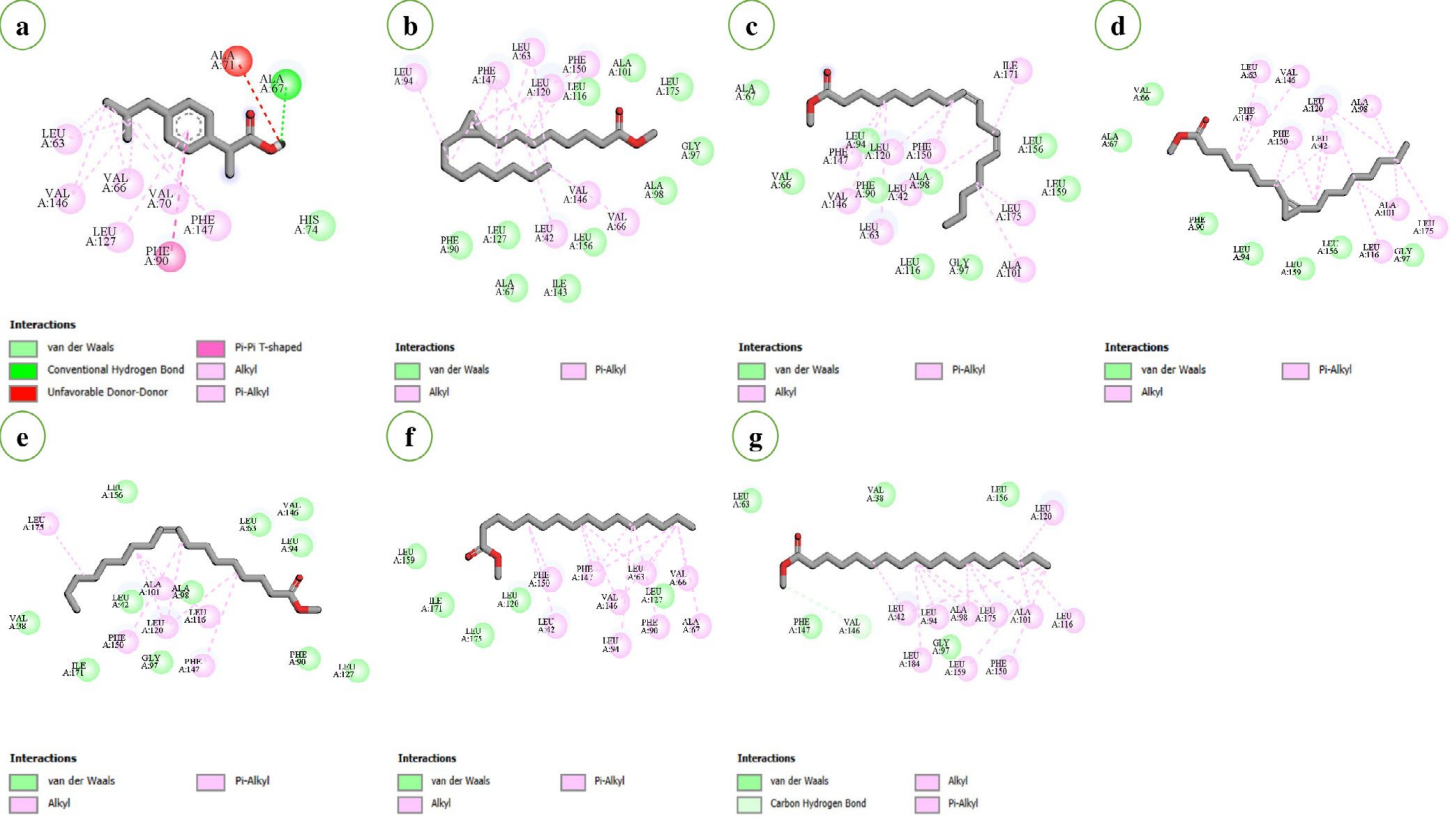
2D binding interactions of TNF‐α with (a) Ibuprofen (standard drug); (b) Cyclopropaneoctanoic acid, 2‐octyl‐, methyl ester; (c) Linoleic acid, methyl ester; (d) Methyl 2‐octylcyclopropene‐1‐heptanoate; (e) Oleic acid, methyl ester; (f) Palmitic acid, methyl ester; (g) Stearic acid, methyl ester.

These moderate docking scores suggest that the observed in vitro activity likely arises from synergistic interactions among multiple compounds rather than from potent single‐target inhibition.

#### 3.7.1 Binding Interactions With *S. aureus* DNA Gyrase (6TTG)

The most active compound, linoleic acid methyl ester, exhibited extensive hydrophobic and van der Waals interactions with multiple residues including ASP57, ARG84, GLY83, GLY85, PRO87, GLU58, ASN54, ASP81, SER55, THR173, ILE51, and LEU103. No classical hydrogen bonds were observed for this compound with 6TTG, suggesting that its binding is primarily driven by hydrophobic packing and van der Waals forces within the active site. For comparison, the reference drug ciprofloxacin formed hydrogen bonds with GLY85 and THR173. A complete summary of all binding interactions is provided in Table S4.

#### 3.7.2 Binding Interactions With *E. coli* DNA Gyrase (1KZN)

Linoleic acid methyl ester formed three hydrogen bonds with ASN46, VAL120, and SER121, in addition to hydrophobic interactions with ALA96, HIS95, MET91, THR165, ASP73, ALA47, and ALA50. The reference drug ciprofloxacin formed hydrogen bonds with ARG76, ARG136, and THR165 (Table S4).

#### 3.7.3 Binding Interactions With COX‐2 (6COX)

Methyl 2‐octylcyclopropene‐1‐heptanoate, which showed the highest binding affinity for COX‐2 (−6.7 kcal/mol), formed a critical hydrogen bond with ARG44 and extensive hydrophobic/van der Waals interactions with GLU46, GLY45, ASN39, GLN461, CYS36, GLY135, ALA156, PRO154, LEU152, GLU465, ARG469, and ASN43. Ibuprofen (reference) bound to ARG469 and GLU465 (Table S4).

#### 3.7.4 Binding Interactions With 5‐LOX (1CYF)

Cyclopropaneoctanoic acid methyl ester, which showed strong binding affinity (−7.7 kcal/mol), formed a hydrogen bond with LEU177 and hydrophobic interactions with MET172, HIS175, TRP191, GLY178, PRO44, VAL47, and PHE266. LEU177 is located near the catalytic site, and its interaction suggests potential competitive inhibition of substrate binding. The reference drug ibuprofen bound to LEU177 via hydrophobic interactions (Table S4).

#### 3.7.5 Binding Interactions With TNF‐α (3F4M)

Methyl 2‐octylcyclopropene‐1‐heptanoate showed strong binding (−6.1 kcal/mol) through hydrophobic interactions with ALA67, VAL66, PHE90, LEU94, LEU159, LEU156, and GLY97. No classical hydrogen bonds were observed for this compound with TNF‐α. These residues are known to be critical for TNF‐α trimerization and receptor binding (Table S4).

### 3.8 Correlation Between In Vitro and In Silico Findings

The in silico docking results were broadly consistent with the in vitro bioactivity data. The ethanolic extract, which contained higher concentrations of fatty acid methyl esters (particularly linoleic and oleic acid derivatives), showed better antibacterial activity against *S. aureus* (MIC: 6.25 mg/mL), and these compounds demonstrated the strongest binding to *S. aureus* DNA gyrase (−5.9 to −7.2 kcal/mol).

However, the aqueous extract, which was not characterized by GC–MS, exhibited superior anti‐inflammatory activity (IC_50_: 129.81 μg/mL) despite the absence of docked fatty acid esters. This suggests that different compound classes (e.g., anthocyanins and phenolic acids) are responsible for the anti‐inflammatory effects. Moderate docking scores (−5 to −7 kcal/mol) indicate that the observed in vitro activity likely arises from synergistic interactions among multiple compounds rather than potent single‐target inhibition.

### 3.9 Computational ADMET Profiling and Drug‐Likeness Analysis

The predicted ADMET properties of the six major compounds are summarized in Tables 11 and 12. Overall, the compounds displayed favorable absorption profiles, with high predicted human intestinal absorption (HIA) and Caco‐2 permeability values. However, most showed high plasma protein binding (PPB > 98%) and limited blood–brain barrier (BBB) penetration, which is typical for lipophilic fatty acid derivatives. All compounds were predicted to inhibit CYP1A2, suggesting the potential for drug–drug interactions. The predicted oral rat LD_50_ values ranged from 3000 to 20,000 mg/kg, classifying them as low toxicity compounds. Drug‐likeness analysis showed that all compounds satisfied Lipinski's Rule of Five but failed some more stringent filters (Ghose, Veber) because of the high number of rotatable bonds.

**TABLE 11:** Predicted ADMET properties of the identified compounds.

Compound	Absorption			Distribution		Metabolism			Excretion		Toxicity	
	Caco‐2 (cm/s)*	HIA*	F%*	PPB (%)*	BBB*	CYP1A2 inhibitor	CYP2C19 inhibitor	CYP2D6 inhibitor	CL* (mL/min/kg)	T1/2 (h)*	hERG*	LD_50_* (mg/kg)
C1*	−5.059	Yes	Yes	98.5	No	Yes	No	No	5.462	0.410	0.367	5000
C2*	−4.999	Yes	Yes	98.1	Yes	Yes	Yes	No	5.901	0.244	0.342	20,000
C3*	−5.021	Yes	Yes	98.4	No	Yes	No	No	5.369	0.335	0.379	3000
C4*	−5.013	Yes	Yes	98.5	No	Yes	No	No	5.279	0.384	0.303	3000
C5*	−5.028	Yes	Yes	98.5	Yes	Yes	No	No	5.278	0.521	0.352	5000
C6*	−5.034	Yes	Yes	99.0	No	Yes	No	No	5.164	0.725	0.424	5000

Abbreviations: BBB*, Blood–brain barrier; C1, Cyclopropaneoctanoic acid, 2‐octyl‐, methyl ester; C2, Linoleic acid methyl ester; C3, Methyl 2‐octylcyclopropene‐1‐heptanoate; C4, Oleic acid methyl ester; C5, Palmitic acid methyl ester; C6, Stearic acid methyl ester; Caco‐2*, Colon adenocarcinoma; CL*, Clearance; F%*, Oral Bioavailability; hERG*, human ether‐a‐go‐go‐related gene; HIA*, Human intestinal absorption; LD_50_*, median lethal dose, indicating the dose at which 50% of test subjects die upon exposure to a compound; PPB*, Plasma protein binding; T 1/2 (h)*, half‐life.

**TABLE 12:** Predicted physicochemical and medicinal chemistry properties.

Compound	Physicochemical property						Medicinal chemistry				
	TPSA* (A^2^)	n‐ROT*	MW* (g/mol)	Log P*	n‐HA*	n‐HD*	Lipinski	Ghose	Veber	Egan	Pfizer
C1*	26.30	16.0	310.51	6.02	2	0	Yes	No	No	No	No
C2*	26.30	15.0	294.47	5.97	2	0	Yes	No	No	No	No
C3*	26.30	15.0	294.47	5.66	2	0	Yes	No	No	No	No
C4*	26.30	16.0	296.49	5.92	2	0	Yes	No	No	No	No
C5*	26.30	15.0	270.45	5.54	2	0	Yes	No	No	Yes	No
C6*	26.30	17.0	298.50	6.24	2	0	Yes	No	No	No	No

Abbreviations: Log P*, logarithm of partition coefficient of compound between n‐octanol and water; MW*, molecular weight; n‐HA*, number of hydrogen bond acceptors; n‐HD*, number of hydrogen bond donors; n‐ROT*, number of rotatable bonds; TPSA*, Topological Polar Surface Area.

### 3.10 Food Science and Nutritional Implications

The findings of this study have direct relevance to the fields of food science and nutrition. The potent antioxidant activity (IC_50_∼290 μg/mL) suggests that *H. sabdariffa* extracts could serve as natural preservatives to inhibit lipid peroxidation in food matrixes. The broad‐spectrum antibacterial activity against foodborne pathogens (including *E. coli* and *S. aureus* ) supports the potential use of roselle extract as a natural food preservative. The non‐toxic profile of the aqueous extract (LD_50_ > 1000 μg/mL) is particularly favorable for food applications. However, direct studies on food matrix preservation (e.g., challenge tests in meat, dairy, or beverage products), sensory evaluation, and stability under processing conditions (temperature, pH, and light) are necessary before commercial application. The deep red color of the extracts, attributable to anthocyanins, presents opportunities for use as natural food colorants.

## 4 Discussion

The present study provides a comprehensive evaluation of *Hibiscus sabdariffa* L. calyx extracts sourced from Libya, integrating phytochemical profiling, multi‐target bioactivity assessment and in silico validation. The key findings revealed that the aqueous extract possessed potent anti‐inflammatory activity (IC_50_: 129.81 μg/mL) and was non‐toxic (LD_50_ > 1000 μg/mL), while the ethanolic extract was rich in fatty acid methyl esters (oleic acid 41.01%, palmitic acid 28.76%) and exhibited bactericidal activity against *S. aureus* (MIC: 6.25 mg/mL). These findings substantiate the traditional use of roselle and highlight the aqueous extract as a promising candidate for functional food and nutraceutical applications (Da‐Costa‐Rocha et al. 2014; Riaz and Chopra 2018; Salem et al. 2022; Singh et al. 2017).

The significantly higher extraction yield of the aqueous extract (51.10%) compared to the ethanolic extract (33.48%) reflects the high water solubility of polar constituents such as polysaccharides, organic acids (hibiscus, citric, and malic acid), and glycosylated anthocyanins (delphinidin‐3‐sambubioside, cyanidin‐3‐sambubioside) (Ojulari et al. 2019; Salem et al. 2022). This observation aligns with traditional preparation methods (infusions and decoctions) and suggests that aqueous extraction is more efficient for recovering the full spectrum of polar bioactive compounds (Abaka et al. 2024; Montalvo‐González et al. 2022). The ethanolic extract, conversely, concentrated lipophilic compounds including fatty acid methyl esters, consistent with previous reports on *H. sabdariffa* seed oils and calyx extracts (Arunasalam et al. 2023; Mohamed et al. 2007). The predominance of oleic acid methyl ester (41.01%) in our extract represents a distinctive feature of the Libyan cultivar. This differential solubility profile directly influences the biological activities observed for each extract (Cissé et al. 2011; Villalobos‐Vega et al. 2023).

Both extracts exhibited broad‐spectrum antibacterial activity against clinically relevant, multidrug‐resistant isolates, with notable solvent‐dependent differences. The ethanolic extract was more effective against Gram‐positive *S. aureus* (MIC: 6.25 mg/mL), while the aqueous extract showed superior activity against Gram‐negative pathogens including *P. aeruginosa* , *K. pneumoniae* , and *P. vulgaris* (MIC: 6.25–12.5 mg/mL). This differential activity can be explained by the distinct chemical compositions of the extracts. The ethanolic extract's effectiveness against *S. aureus* likely arises from its high content of fatty acid methyl esters, which can disrupt bacterial cell membranes by intercalating into the lipid bilayer, increasing permeability, and causing leakage of cellular contents (Desbois and Smith 2010). In contrast, the aqueous extract's remarkable activity against Gram‐negative pathogens suggests the presence of water‐soluble antimicrobials such as phenolic acids (gallic acid, protocatechuic acid, hibiscus acid), which can chelate iron, generate reactive oxygen species, and interfere with quorum sensing systems (Bakrim et al. 2022; Zhang et al. 2021). Polysaccharides and saponins may also contribute by disrupting membrane integrity.

The MBC/MIC ratios (≤ 2 for all combinations) confirmed bactericidal activity, which is clinically advantageous for treating infections in immunocompromised patients. Importantly, isolates resistant to conventional antibiotics remained susceptible to the extracts, suggesting that *H. sabdariffa* may employ different or multi‐target mechanisms that circumvent existing resistance pathways (Vaou et al. 2021). This is a crucial advantage in the era of antimicrobial resistance, where the development of new therapeutic alternatives is urgently needed (Murray et al. 2022; Organization 2014).

The observed MIC values (6.25–25 mg/mL) are substantially higher than those of purified antibiotics (typically 0.1–2 μg/mL for susceptible strains). This is expected for crude plant extracts, which contain a mixture of active and inert compounds. Direct comparison with standard antibiotics is therefore not appropriate; rather, the activity should be interpreted as promising for a crude preparation, particularly against multidrug‐resistant isolates. Future fractionation and bioassay‐guided isolation may yield more potent compounds.

The comparable antioxidant activities of both extracts (IC_50_: 283.6 vs. 292.1 μg/mL) indicate that radical‐scavenging compounds were efficiently extracted by both polar and semi‐polar solvents. The primary antioxidants in *H. sabdariffa* are anthocyanins, phenolic acids, and flavonoids (Hapsari and Setyaningsih 2021). These compounds neutralize free radicals through hydrogen atom transfer or single‐electron transfer mechanisms, thereby terminating radical chain reactions and preventing oxidative damage (Munteanu and Apetrei 2021). Although less potent than pure gallic acid, the activity of the extracts is biologically significant and compares favorably with other medicinal plants used as functional beverages (Munteanu and Apetrei 2021). The antioxidant capacity supports the traditional use of roselle tea for mitigating oxidative stress, a key factor in chronic inflammatory diseases, cardiovascular disorders, and aging (Mengozzi et al. 2021; Somaratne et al. 2025).

The most striking finding of this study was the exceptional anti‐inflammatory activity of the aqueous extract (IC_50_: 129.81 μg/mL), which was approximately threefold more potent than that of the ethanolic extract (IC_50_: 379.46 μg/mL, *p* = 0.008). This challenges the conventional preference for organic solvents in the extraction of anti‐inflammatory phytochemicals, highlighting the importance of solvent selection based on traditional preparation methods (Metoui et al. 2022; Truong et al. 2019). The albumin denaturation assay is an established in vitro model for preliminary anti‐arthritic screening (Uttra 2017). Inhibition of denaturation stabilizes proteins and prevents the release of inflammatory mediators. However, confirmation of anti‐inflammatory activity requires enzymatic (COX‐2, 5‐LOX inhibition) and cellular (e.g., LPS‐stimulated macrophage nitric oxide or cytokine production) assays. The molecular docking results presented here are predictive and suggest potential interactions with these targets, but do not constitute pharmacological validation. The superior activity of the aqueous extract suggests that polar constituents, particularly polysaccharides, saponins, and highly polar phenolic acids, may contribute to protein stabilization, though this hypothesis requires direct testing (Kao et al. 2009; Sies and Jones 2020).

Molecular docking provided mechanistic insights into the anti‐inflammatory potential of the constituents of the ethanolic extract. Cyclopropaneoctanoic acid methyl ester showed strong binding to 5‐lipoxygenase (−7.7 kcal/mol), comparable to ibuprofen (−7.9 kcal/mol). 5‐Lipoxygenase is a key enzyme in the biosynthesis of leukotrienes, which are potent inflammatory mediators involved in asthma, arthritis, and inflammatory bowel disease (Fiorucci et al. 2001). Inhibition of 5‐lipoxygenase, together with cyclooxygenase‐2 inhibition (observed for methyl 2‐octylcyclopropene‐1‐heptanoate, −6.7 kcal/mol), suggests the potential for dual inhibition of the arachidonic acid cascade, which reduces both prostaglandins and leukotrienes. This dual inhibition profile is clinically advantageous because selective cyclooxygenase‐2 inhibitors have been associated with cardiovascular side effects, whereas dual inhibitors may offer improved safety profiles (Fiorucci et al. 2001). However, the absence of these fatty acid esters in the aqueous extract suggests that different compound classes are responsible for their anti‐inflammatory activity. Anthocyanins have been shown to inhibit NF‐κB activation and reduce TNF‐α, IL‐1β, and IL‐6 production in LPS‐stimulated macrophages (Kao et al. 2009).

The differential cytotoxicity between the aqueous (non‐toxic, LD_50_ > 1000 μg/mL) and ethanolic (moderately toxic, LD_50_: 345.5 μg/mL) extracts is a critical finding. The brine shrimp lethality assay correlates well with mammalian cytotoxicity in approximately 70% of cases (Parra et al. 2001). The non‐toxic profile of the aqueous extract supports its long‐term use as a functional beverage, whereas the moderate toxicity of the ethanolic extract necessitates caution (Rosli et al. 2026). The toxicity of the ethanolic extract may be attributed to the concentration of lipophilic compounds, including fatty acid methyl esters and potentially other secondary metabolites not identified by GC–MS (Al‐Rubaye et al. 2017).

It is important to acknowledge that the GC–MS method preferentially detects volatile and semi‐volatile constituents. Polar compounds such as anthocyanins (e.g., delphinidin‐3‐sambubioside), phenolic acids (e.g., hibiscus acid, chlorogenic acid), and other polyphenolic glycosides widely recognized as principal bioactive constituents of *H. sabdariffa* are not amenable to GC–MS analysis without derivatization and therefore were not characterized in this study. Additionally, the detection of fatty acid methyl esters raises the possibility of partial transesterification during ethanolic extraction, although these esters have also been reported as naturally occurring constituents in *H. sabdariffa* calyces by other researchers. Future studies employing LC–MS/MS or HPLC‐DAD are necessary to fully profile the polar phytochemical composition, particularly for the aqueous extract which exhibited potent anti‐inflammatory activity.

In silico ADMET predictions provide valuable guidance for future drug development. The high predicted HIA and Caco‐2 permeability suggest good oral bioavailability, supporting the traditional oral administration of Roselle tea (Daina et al. 2017). However, the high PPB (> 98%) may reduce free drug concentrations, and predicted CYP1A2 inhibition raises the possibility of drug–drug interactions. The favorable predicted oral LD_50_ values (3000–20,000 mg/kg) and compliance with Lipinski's Rule of Five support the further development of these compounds as drug candidates or nutraceuticals (Daina et al. 2017).

The findings are broadly consistent with previous reports but show notable regional differences. The IC_50_ values for antioxidant activity (283–292 μg/mL) were slightly higher than those reported for Egyptian roselle (150–200 μg/mL) but lower than those for Nigerian roselle (350–450 μg/mL) (Okwanya et al. 1970; Osman et al. 2008). These regional variations likely reflect differences in growing conditions, harvest time, and post‐harvest processing (Balois‐Morales et al. 2016). The potent anti‐inflammatory activity of the aqueous extract (IC_50_: 129.81 μg/mL) compares favorably with previous reports (IC_50_ range: 150–300 μg/mL) and may represent a unique characteristic of the Libyan cultivar.

The in silico docking results were broadly consistent with the in vitro data. The ethanolic extract, rich in fatty acid methyl esters, exhibited better activity against *S. aureus* , and these compounds demonstrated the strongest binding to DNA gyrase. Moderate docking scores (−5 to −7 kcal/mol) indicate that the observed in vitro activity likely arises from synergistic interactions among multiple compounds rather than potent single‐target inhibition, consistent with the traditional use of whole‐plant extracts (Vaou et al. 2021).

The findings of this study also have direct relevance to food science and nutrition. The potent antioxidant activity (IC_50_∼290 μg/mL) suggests that *H. sabdariffa* extracts could serve as natural preservatives to inhibit lipid peroxidation in food matrices (Duggirala et al. 2024; Jabeur et al. 2017). The broad‐spectrum antibacterial activity against foodborne pathogens (including *E. coli* and *S. aureus* ) supports the potential use of roselle extracts as natural food preservatives. Previous studies have successfully incorporated *H. sabdariffa* by‐products into food products such as crackers, demonstrating increased total phenolic content (from 5.99 to 17.57 mg/g) and total flavonoid content (from 49.36 to 104.63 mg/g), with DPPH radical scavenging activity increasing twofold (Ahmed and Abozed 2015). The non‐toxic profile of the aqueous extract (LD_50_ > 1000 μg/mL) is particularly favorable for food applications. The deep red color of the extracts, attributable to anthocyanins, also presents opportunities as natural food colorants. However, direct studies on food matrix preservation (e.g., challenge tests in meat, dairy, or beverage products), sensory evaluation, and stability under processing conditions (temperature, pH, light) are necessary before commercial application (Duggirala et al. 2024; Kizzie‐Hayford et al. 2024; Ndoye et al. 2025; Ngo et al. 2024; Sipahli et al. 2017; Violet and Suryoprabowo 2025).

This study has several limitations that warrant acknowledgment. First, the use of crude extracts precludes definitive attribution of bioactivity to specific compounds.

Second, the brine shrimp lethality assay, while useful for preliminary screening, does not replace mammalian cytotoxicity assays (e.g., MTT on Vero, HepG2, or RAW 264.7 cells) or in vivo toxicity studies. Third, molecular docking was restricted to the compounds identified by GC–MS (primarily fatty acid derivatives), and the potent anti‐inflammatory activity of the aqueous extract likely mediated by polar anthocyanins and phenolic acids remains to be characterized by LC–MS/MS. Fourth, the anti‐inflammatory evaluation relied solely on the protein denaturation inhibition model without confirmation via enzymatic (COX/LOX) or cellular (macrophage NO inhibition) assays. Docking results should be considered hypothesis‐generating rather than confirmatory. Fifth, the plant material was collected from a single location (Al‐Bayda, Libya) during one growing season (May–July 2024), which may limit generalizability due to batch‐to‐batch variability across different seasons or geographical locations. Sixth, time‐kill kinetics studies were not performed; therefore, the bactericidal rate and duration of action of the extracts remain unknown. Seventh, the study did not investigate potential synergistic interactions between the extracts and conventional antibiotics, which could be valuable for combating multidrug‐resistant infections.

Future studies should address these gaps through: (1) liquid chromatography–tandem mass spectrometry characterization of the aqueous extract to identify its bioactive polar compounds; (2) in vivo efficacy studies in animal models of inflammation; (3) mammalian cell line cytotoxicity assays to confirm the safety profile; (4) formulation development for topical and oral applications; (5) checkerboard synergy studies with conventional antibiotics against multidrug‐resistant isolates; (6) time‐kill kinetics studies to determine bactericidal rates; (7) stability studies under various processing conditions for food applications; and (8) clinical trials for specific indications. Addressing these gaps will strengthen the translational potential of Libyan *H. sabdariffa* following confirmatory in vivo studies and mammalian toxicity testing and functional food.

## 5 Conclusion

This integrated study provides robust scientific evidence supporting the therapeutic and nutraceutical potential of *Hibiscus sabdariffa* L. calyces sourced from Libya. The aqueous extract emerged as a particularly promising candidate due to its potent anti‐inflammatory activity (IC_50_: 129.81 μg/mL) and favorable preliminary toxicity profile in the brine shrimp model (LD_50_ > 1000 μg/mL), while the ethanolic extract, rich in fatty acid methyl esters, demonstrated significant bactericidal activity against multidrug‐resistant *S. aureus* (MIC: 6.25 mg/mL) with moderate toxicity. Both extracts exhibited comparable antioxidant activity (IC_50_: 283.6–292.1 μg/mL). Molecular docking revealed moderate binding affinities to bacterial DNA gyrase and anti‐inflammatory targets, with ADMET predictions indicating favorable pharmacokinetic profiles despite potential CYP1A2 inhibition. These findings substantiate the traditional use of roselle and support further investigation toward potential development as a standardized herbal preparation or functional food ingredient, particularly the non‐toxic aqueous extract for functional beverage formulations and natural food preservatives.

## Author Contributions

**Zuhir S. Mussa Akrim:** writing – review and editing, writing – original draft, software. **Ahmed Saeed Kabbashi:** conceptualization, investigation, writing – original draft, methodology, validation, visualization, writing – review and editing, software, formal analysis, project administration, supervision, resources. **Maryam Mohammed Ibrahim:** conceptualization, investigation, funding acquisition, writing – original draft, methodology, validation, software, formal analysis, resources, data curation. **Mona Rafea Mosa:** conceptualization, investigation, funding acquisition, writing – original draft, methodology, validation, software, formal analysis, data curation, resources. **Ahmed Ali Mustafa:** investigation, writing – original draft, writing – review and editing. **Mohammed B. Suliman:** investigation, writing – original draft, writing – review and editing, formal analysis. **Sanadelaslam S. A. El‐Hddad:** writing – review and editing, formal analysis. **Rama Burhan Hasan:** conceptualization, investigation, funding acquisition, writing – original draft, methodology, validation, software, formal analysis, data curation, resources. **Esraa Radwan Ibrahim:** conceptualization, investigation, funding acquisition, writing – original draft, methodology, validation, software, formal analysis, data curation, resources.

## Funding

The authors have nothing to report.

## Conflicts of Interest

The authors declare no conflicts of interest.

## Supporting information


**Figure S1:** 3D binding interactions of DNA gyrase (6TTG) with (a) Ciprofloxacin (standard drug); (b) Cyclopropaneoctanoic acid, 2‐octyl‐, methyl ester; (c) Linoleic acid, methyl ester; (d) Methyl 2‐octylcyclopropene‐1‐heptanoate; (e) Oleic acid, methyl ester; (f) Palmitic acid, methyl ester; (g) Stearic acid, methyl ester.
**Figure S2:** 3D binding interactions of DNA gyrase (1KZN) with (a) Ciprofloxacin (standard drug); (b) Cyclopropaneoctanoic acid, 2‐octyl‐, methyl ester; (c) Linoleic acid, methyl ester; (d) Methyl 2‐octylcyclopropene‐1‐heptanoate; (e) Oleic acid, methyl ester; (f) Palmitic acid, methyl ester; (g) Stearic acid, methyl ester.
**Figure S3:** 3D binding interactions of COX‐2 with (a) Ibuprofen (standard drug); (b) Cyclopropaneoctanoic acid, 2‐octyl‐, methyl ester; (c) Linoleic acid, methyl ester; (d) Methyl 2‐octylcyclopropene‐1‐heptanoate; (e) Oleic acid, methyl ester; (f) Palmitic acid, methyl ester; (g) Stearic acid, methyl ester.
**Figure S4:** 3D binding interactions of 5‐LOX with (a) Ibuprofen (standard drug); (b) Cyclopropaneoctanoic acid, 2‐octyl‐, methyl ester; (c) Linoleic acid, methyl ester; (d) Methyl 2‐octylcyclopropene‐1‐heptanoate; (e) Oleic acid, methyl ester; (f) Palmitic acid, methyl ester; and (g) Stearic acid, methyl ester.
**Figure S5:** 3D binding interactions of TNF‐α with (a) Ibuprofen (standard drug); (b) Cyclopropaneoctanoic acid, 2‐octyl‐, methyl ester; (c) Linoleic acid, methyl ester; (d) Methyl 2‐octylcyclopropene‐1‐heptanoate; (e) Oleic acid, methyl ester; (f) Palmitic acid, methyl ester; (g) Stearic acid, methyl ester.
**Figure S6:** 2D binding interactions of DNA gyrase (6TTG) with (a) ciprofloxacin; (b) Linoleic acid; (c) Oleic acid; (d) Palmitic acid; (e) Stearic acid; (f) Cyclopropaneoctanoic acid, 2‐octyl‐; (g) Methyl 2‐octylcyclopropene‐1‐heptanoate (free acid form).
**Figure S7:** 3D binding interactions of DNA gyrase (6TTG) with (a) ciprofloxacin; (b) Linoleic acid; (c) Oleic acid; (d) Palmitic acid; (e) Stearic acid; (f) Cyclopropaneoctanoic acid, 2‐octyl‐; (g) Methyl 2‐octylcyclopropene‐1‐heptanoate (free acid form).
**Figure S8:** 2D binding interactions of DNA gyrase (1KZN) with (a) ciprofloxacin; (b) Linoleic acid; (c) Oleic acid; (d) Palmitic acid; (e) Stearic acid; (f) Cyclopropaneoctanoic acid, 2‐octyl‐; (g) Methyl 2‐octylcyclopropene‐1‐heptanoate (free acid form).
**Figure S9:** 3D binding interactions of DNA gyrase (1KZN) with (a) ciprofloxacin; (b) Linoleic acid; (c) Oleic acid; (d) Palmitic acid; (e) Stearic acid; (f) Cyclopropaneoctanoic acid, 2‐octyl‐; (g) Methyl 2‐octylcyclopropene‐1‐heptanoate (free acid form).
**Figure S10:** 2D binding interactions of COX‐2 with (a) ciprofloxacin; (b) Linoleic acid; (c) Oleic acid; (d) Palmitic acid; (e) Stearic acid; (f) Cyclopropaneoctanoic acid, 2‐octyl‐; (g) Methyl 2‐octylcyclopropene‐1‐heptanoate (free acid form).
**Figure S11:** 3D binding interactions of COX‐2 with (a) ciprofloxacin; (b) Linoleic acid; (c) Oleic acid; (d) Palmitic acid; (e) Stearic acid; (f) Cyclopropaneoctanoic acid, 2‐octyl‐; (g) Methyl 2‐octylcyclopropene‐1‐heptanoate (free acid form).
**Figure S12:** 2D binding interactions of 5‐LOX with (a) ciprofloxacin; (b) Linoleic acid; (c) Oleic acid; (d) Palmitic acid; (e) Stearic acid; (f) Cyclopropaneoctanoic acid, 2‐octyl‐; (g) Methyl 2‐octylcyclopropene‐1‐heptanoate (free acid form).
**Figure S13:** 3D binding interactions of 5‐LOX with (a) ciprofloxacin; (b) Linoleic acid; (c) Oleic acid; (d) Palmitic acid; (e) Stearic acid; (f) Cyclopropaneoctanoic acid, 2‐octyl‐; (g) Methyl 2‐octylcyclopropene‐1‐heptanoate (free acid form).
**Figure S14:** 2D binding interactions of TNF‐α with (a) ciprofloxacin; (b) Linoleic acid; (c) Oleic acid; (d) Palmitic acid; (e) Stearic acid; (f) Cyclopropaneoctanoic acid, 2‐octyl‐; (g) Methyl 2‐octylcyclopropene‐1‐heptanoate (free acid form).
**Figure S15:** 3D binding interactions of TNF‐α with (a) ciprofloxacin; (b) Linoleic acid; (c) Oleic acid; (d) Palmitic acid; (e) Stearic acid; (f) Cyclopropaneoctanoic acid, 2‐octyl‐; (g) Methyl 2‐octylcyclopropene‐1‐heptanoate (free acid form).
**Table S1:** Docking scores of free fatty acids against bacterial DNA gyrase (6TTG, 1KZN).
**Table S2:** Docking scores of free fatty acids against anti‐inflammatory targets (COX‐2, 5‐LOX, TNF‐α).
**Table S3:** Redocking validation results (RMSD values).
**Table S4:** Summary of key binding interactions for the most active compounds against all five protein targets.

## Data Availability

The data supporting the findings of this study are available from the corresponding author (Dr. Ahmed Saeed Kabbashi) upon reasonable request.
