# Supplementary material for: Comprehensive Phytochemical Profiling and Multi‐Target Biological Evaluation of Hibiscus sabdariffa L. Calyx Extracts From Libya: A Promising Functional Food Candidate With Antibacterial, Antioxidant, Anti‐Inflammatory, and Cytotoxic Properties: Validation Through In Silico Approaches
Source: Food Sci Nutr. 2026 Jun 30;14(7):e72056. doi: 10.1002/fsn3.72056 (PMC13316456; doi:10.1002/fsn3.72056)
Supplement: Supplementary file 1 — Figure S1: 3D binding interactions of DNA gyrase (6TTG) with (a) Ciprofloxacin (standard drug); (b) Cyclopropaneoctanoic acid, 2‐octyl‐, methyl ester; (c) Linoleic acid, methyl ester; (d) Methyl 2‐octylcyclopropene‐1‐heptanoate; (e) Oleic acid, methyl ester; (f) Palmitic acid, methyl ester; (g) Stearic acid, methyl ester. Figure S2: 3D binding interactions of DNA gyrase (1KZN) with (a) Ciprofloxacin (standard drug); (b) Cyclopropaneoctanoic acid, 2‐octyl‐, methyl ester; (c) Linoleic acid, methyl ester; (d) Methyl 2‐octylcyclopropene‐1‐heptanoate; (e) Oleic acid, methyl ester; (f) Palmitic acid, methyl ester; (g) Stearic acid, methyl ester. Figure S3: 3D binding interactions of COX‐2 with (a) Ibuprofen (standard drug); (b) Cyclopropaneoctanoic acid, 2‐octyl‐, methyl ester; (c) Linoleic acid, methyl ester; (d) Methyl 2‐octylcyclopropene‐1‐heptanoate; (e) Oleic acid, methyl ester; (f) Palmitic acid, methyl ester; (g) Stearic acid, methyl ester. Figure S4: 3D binding interactions of 5‐LOX with (a) Ibuprofen (standard drug); (b) Cyclopropaneoctanoic acid, 2‐octyl‐, methyl ester; (c) Linoleic acid, methyl ester; (d) Methyl 2‐octylcyclopropene‐1‐heptanoate; (e) Oleic acid, methyl ester; (f) Palmitic acid, methyl ester; and (g) Stearic acid, methyl ester. Figure S5: 3D binding interactions of TNF‐α with (a) Ibuprofen (standard drug); (b) Cyclopropaneoctanoic acid, 2‐octyl‐, methyl ester; (c) Linoleic acid, methyl ester; (d) Methyl 2‐octylcyclopropene‐1‐heptanoate; (e) Oleic acid, methyl ester; (f) Palmitic acid, methyl ester; (g) Stearic acid, methyl ester. Figure S6: 2D binding interactions of DNA gyrase (6TTG) with (a) ciprofloxacin; (b) Linoleic acid; (c) Oleic acid; (d) Palmitic acid; (e) Stearic acid; (f) Cyclopropaneoctanoic acid, 2‐octyl‐; (g) Methyl 2‐octylcyclopropene‐1‐heptanoate (free acid form). Figure S7: 3D binding interactions of DNA gyrase (6TTG) with (a) ciprofloxacin; (b) Linoleic acid; (c) Oleic acid; (d) Palmitic acid; (e) Stearic acid; (f) Cyclopropaneo [file FSN3-14-e72056-s001.docx]

**Supplementary Material**

**Comprehensive Phytochemical Profiling and Multi-Target Biological Evaluation of *Hibiscus sabdariffa* L. Calyx Extracts from Libya: A Promising Functional Food Candidate with Antibacterial, Antioxidant, Anti-inflammatory, and Cytotoxic Properties: Validation Through *In Silico* Approaches**

Ahmed Saeed Kabbashi, Zuhir S. Mussa Akrim, Mohammed B. Suliman, Sanadelaslam S. A. El-Hddad, Ahmed A. Mustafa, Maryam Mohammed Ibrahim, Esraa Radwan Ibrahim, Mona Rafea Mosa, Rama Burhan Hasan

**Table of Contents**

**Table S1:** Docking scores of free fatty acids against bacterial DNA gyrase (6TTG, 1KZN)

**Table S2:** Docking scores of free fatty acids against anti-inflammatory targets (COX-2, 5-LOX, TNF-α)

**Table S3:** Redocking validation results (RMSD values)

**Table S4:** Summary of key binding interactions for the most active compounds against all five protein targets

**Figure of Contents**

**Figure S1.** 3D binding interactions of DNA gyrase (6TTG) with (a) Ciprofloxacin (standard drug); (b) Cyclopropaneoctanoic acid, 2-octyl-, methyl ester; (c) Linoleic acid, methyl ester; (d) Methyl 2-octylcyclopropene-1-heptanoate; (e) Oleic acid, methyl ester; (f) Palmitic acid, methyl ester; (g) Stearic acid, methyl ester.

**Figure S2.** 3D binding interactions of DNA gyrase (1KZN) with (a) Ciprofloxacin (standard drug); (b) Cyclopropaneoctanoic acid, 2-octyl-, methyl ester; (c) Linoleic acid, methyl ester; (d) Methyl 2-octylcyclopropene-1-heptanoate; (e) Oleic acid, methyl ester; (f) Palmitic acid, methyl ester; (g) Stearic acid, methyl ester.

**Figure S3.** 3D binding interactions of COX-2 with (a) Ibuprofen (standard drug); (b) Cyclopropaneoctanoic acid, 2-octyl-, methyl ester; (c) Linoleic acid, methyl ester; (d) Methyl 2-octylcyclopropene-1-heptanoate; (e) Oleic acid, methyl ester; (f) Palmitic acid, methyl ester; (g) Stearic acid, methyl ester.

**Figure S4.** 3D binding interactions of 5-LOX with (a) Ibuprofen (standard drug); (b) Cyclopropaneoctanoic acid, 2-octyl-, methyl ester; (c) Linoleic acid, methyl ester; (d) Methyl 2-octylcyclopropene-1-heptanoate; (e) Oleic acid, methyl ester; (f) Palmitic acid, methyl ester; and (g) Stearic acid, methyl ester.

**Figure S5.** 3D binding interactions of TNF-α with (a) Ibuprofen (standard drug); (b) Cyclopropaneoctanoic acid, 2-octyl-, methyl ester; (c) Linoleic acid, methyl ester; (d) Methyl 2-octylcyclopropene-1-heptanoate; (e) Oleic acid, methyl ester; (f) Palmitic acid, methyl ester; (g) Stearic acid, methyl ester.

**Figure S6:** 2D binding interactions of DNA gyrase (6TTG) with **(a)** ciprofloxacin; **(b)** Linoleic acid; **(c)** Oleic acid; **(d)** Palmitic acid; **(e)** Stearic acid; **(f)** Cyclopropaneoctanoic acid, 2-octyl-; **(g)** Methyl 2-octylcyclopropene-1-heptanoate (free acid form).

**Figure S7:** 3D binding interactions of DNA gyrase (6TTG) with **(a)** ciprofloxacin; **(b)** Linoleic acid; **(c)** Oleic acid; **(d)** Palmitic acid; **(e)** Stearic acid; **(f)** Cyclopropaneoctanoic acid, 2-octyl-; **(g)** Methyl 2-octylcyclopropene-1-heptanoate (free acid form).

**Figure S8:** 2D binding interactions of DNA gyrase (1KZN) with **(a)** ciprofloxacin; **(b)** Linoleic acid; **(c)** Oleic acid; **(d)** Palmitic acid; **(e)** Stearic acid; **(f)** Cyclopropaneoctanoic acid, 2-octyl-; **(g)** Methyl 2-octylcyclopropene-1-heptanoate (free acid form).

**Figure S9:** 3D binding interactions of DNA gyrase (1KZN) with **(a)** ciprofloxacin; **(b)** Linoleic acid; **(c)** Oleic acid; **(d)** Palmitic acid; **(e)** Stearic acid; **(f)** Cyclopropaneoctanoic acid, 2-octyl-; **(g)** Methyl 2-octylcyclopropene-1-heptanoate (free acid form).

**Figure S10:** 2D binding interactions of COX-2 with **(a)** ciprofloxacin; **(b)** Linoleic acid; **(c)** Oleic acid; **(d)** Palmitic acid; **(e)** Stearic acid; **(f)** Cyclopropaneoctanoic acid, 2-octyl-; **(g)** Methyl 2-octylcyclopropene-1-heptanoate (free acid form).

**Figure S11:** 3D binding interactions of COX-2 with **(a)** ciprofloxacin; **(b)** Linoleic acid; **(c)** Oleic acid; **(d)** Palmitic acid; **(e)** Stearic acid; **(f)** Cyclopropaneoctanoic acid, 2-octyl-; **(g)** Methyl 2-octylcyclopropene-1-heptanoate (free acid form).

**Figure S12:** 2D binding interactions of 5-LOX with **(a)** ciprofloxacin; **(b)** Linoleic acid; **(c)** Oleic acid; **(d)** Palmitic acid; **(e)** Stearic acid; **(f)** Cyclopropaneoctanoic acid, 2-octyl-; **(g)** Methyl 2-octylcyclopropene-1-heptanoate (free acid form).

**Figure S13:** 3D binding interactions of 5-LOX with **(a)** ciprofloxacin; **(b)** Linoleic acid; **(c)** Oleic acid; **(d)** Palmitic acid; **(e)** Stearic acid; **(f)** Cyclopropaneoctanoic acid, 2-octyl-; **(g)** Methyl 2-octylcyclopropene-1-heptanoate (free acid form).

**Figure S14:** 2D binding interactions of TNF-α with **(a)** ciprofloxacin; **(b)** Linoleic acid; **(c)** Oleic acid; **(d)** Palmitic acid; **(e)** Stearic acid; **(f)** Cyclopropaneoctanoic acid, 2-octyl-; **(g)** Methyl 2-octylcyclopropene-1-heptanoate (free acid form).

**Figure S15:** 3D binding interactions of TNF-α with **(a)** ciprofloxacin; **(b)** Linoleic acid; **(c)** Oleic acid; **(d)** Palmitic acid; **(e)** Stearic acid; **(f)** Cyclopropaneoctanoic acid, 2-octyl-; **(g)** Methyl 2-octylcyclopropene-1-heptanoate (free acid form).


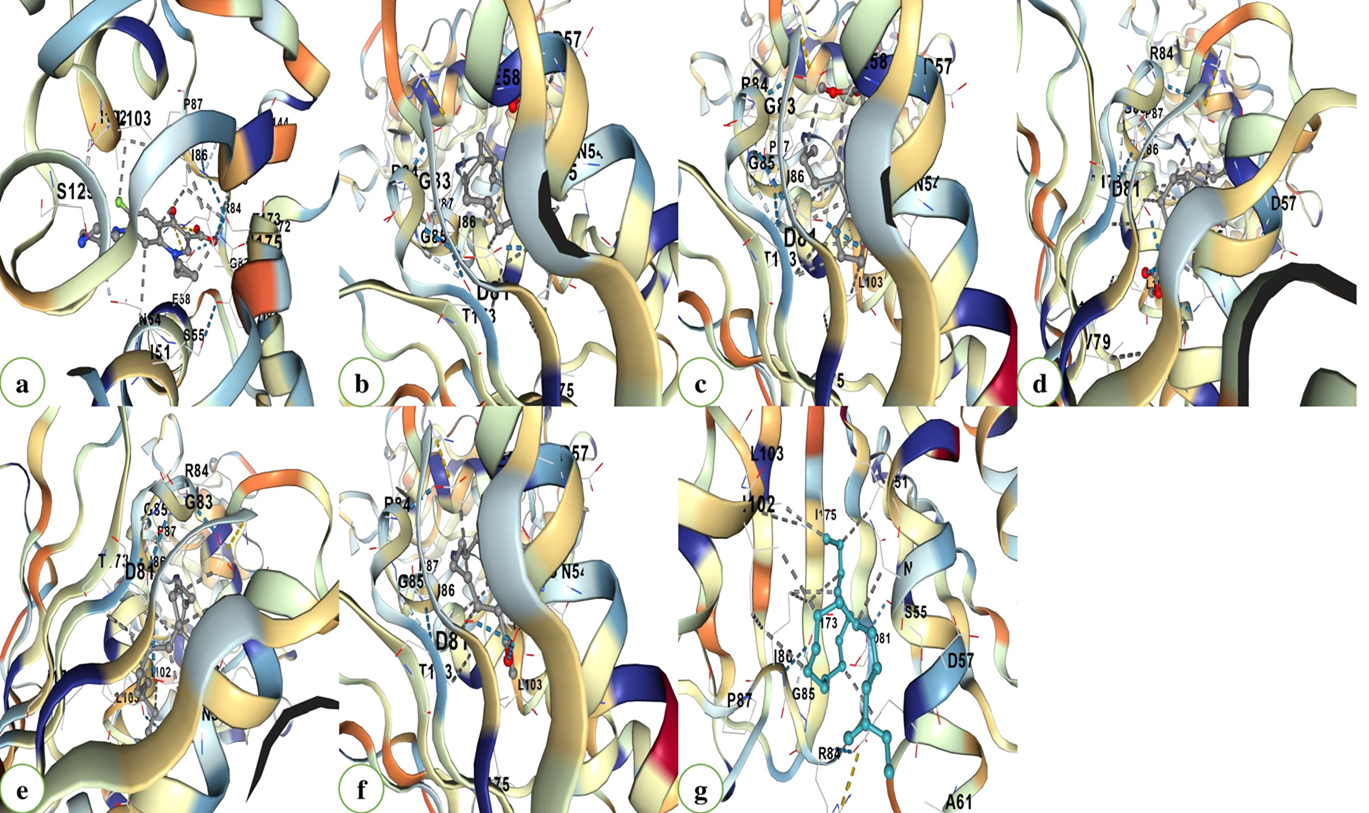


**Figure S1.** 3D binding interactions of DNA gyrase (6TTG) with **(a)** Ciprofloxacin (standard drug); **(b)** Cyclopropaneoctanoic acid, 2-octyl-, methyl ester; **(c)** Linoleic acid, methyl ester; **(d)** Methyl 2-octylcyclopropene-1-heptanoate; **(e)** Oleic acid, methyl ester; **(f)** Palmitic acid, methyl ester; **(g)** Stearic acid, methyl ester.


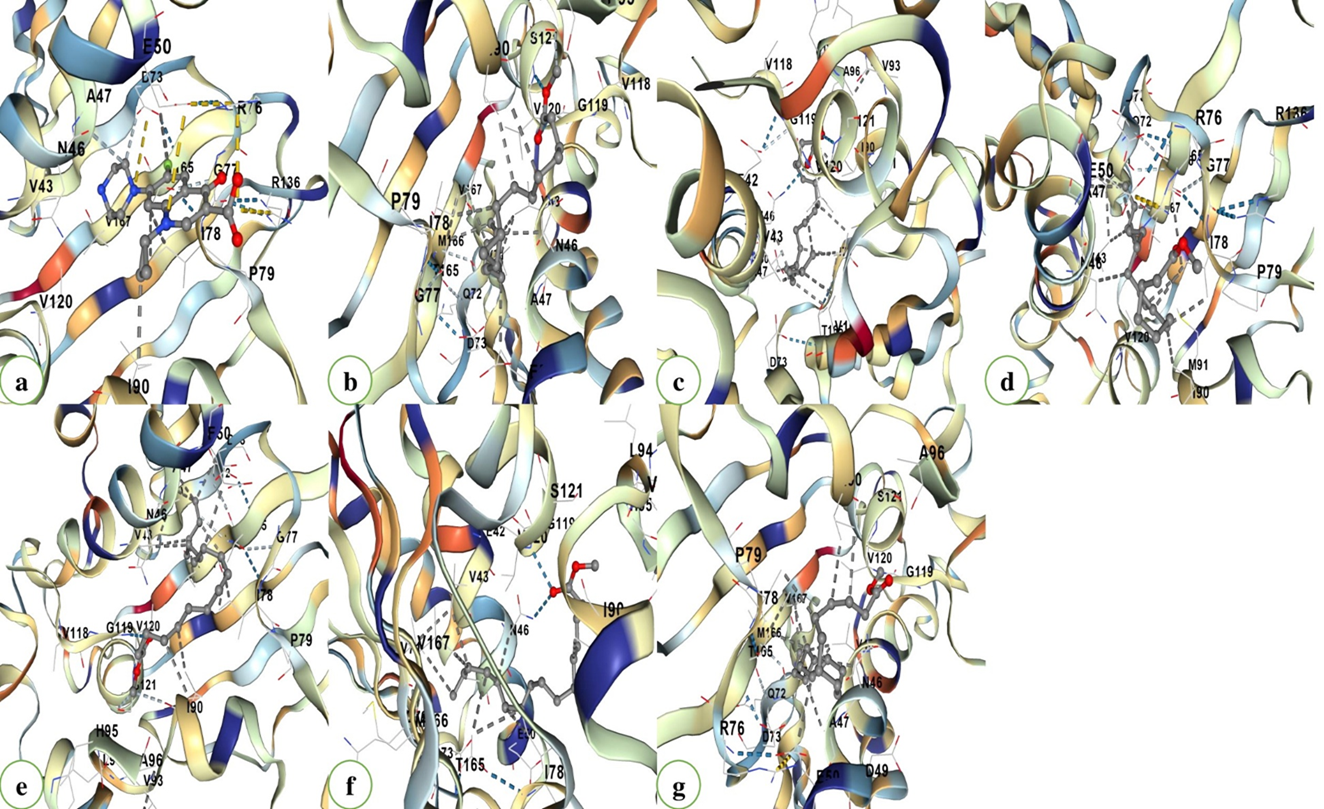


**Figure S2.** 3D binding interactions of DNA gyrase (1KZN) with **(a)** Ciprofloxacin (standard drug); **(b)** Cyclopropaneoctanoic acid, 2-octyl-, methyl ester; **(c)** Linoleic acid, methyl ester; **(d)** Methyl 2-octylcyclopropene-1-heptanoate; **(e)** Oleic acid, methyl ester; **(f)** Palmitic acid, methyl ester; **(g)** Stearic acid, methyl ester.


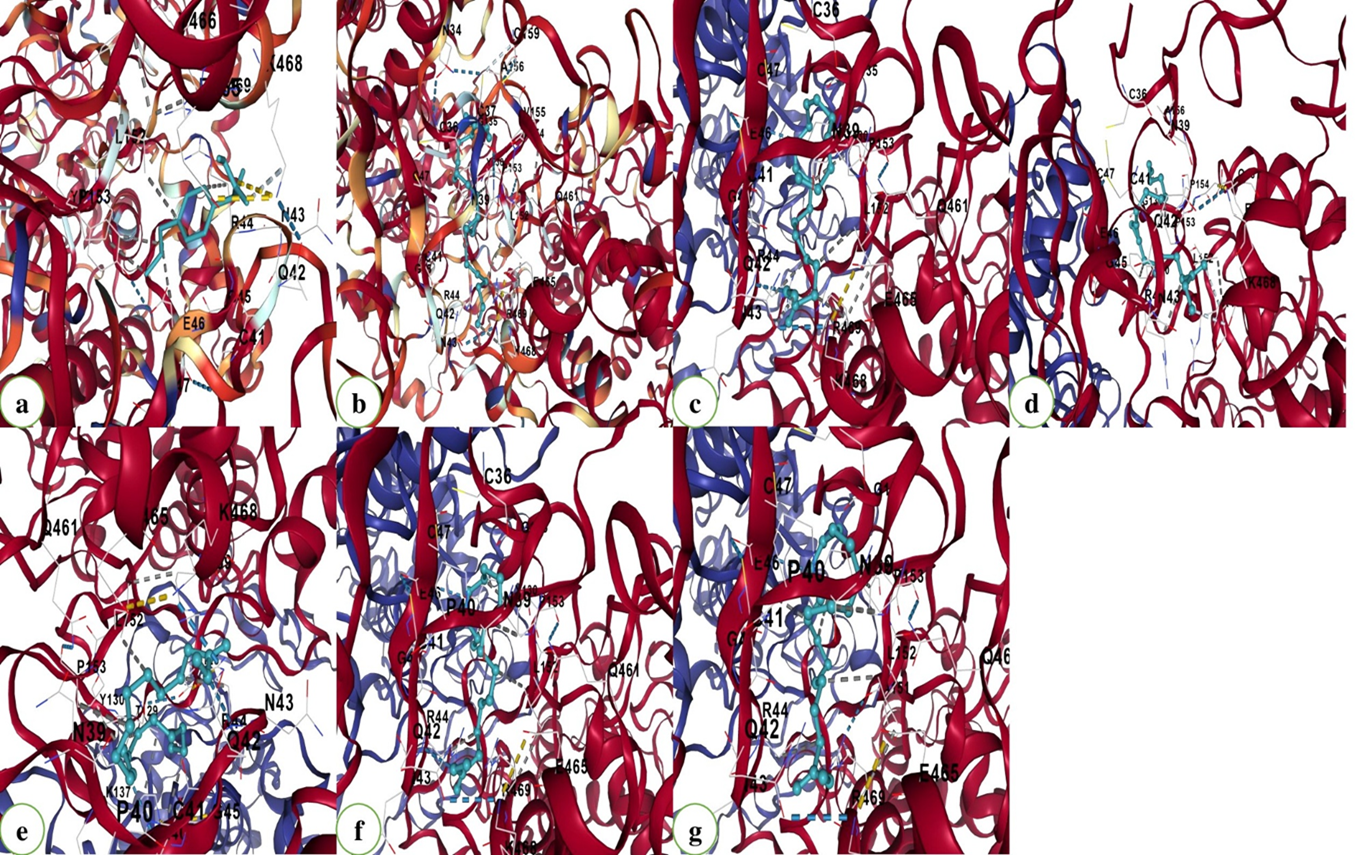
**Figure S3.** 3D binding interactions of COX-2 with **(a)** Ibuprofen (standard drug); **(b)** Cyclopropaneoctanoic acid, 2-octyl-, methyl ester; **(c)** Linoleic acid, methyl ester; **(d)** Methyl 2-octylcyclopropene-1-heptanoate; **(e)** Oleic acid, methyl ester; **(f)** Palmitic acid, methyl ester; **(g)** Stearic acid, methyl ester.


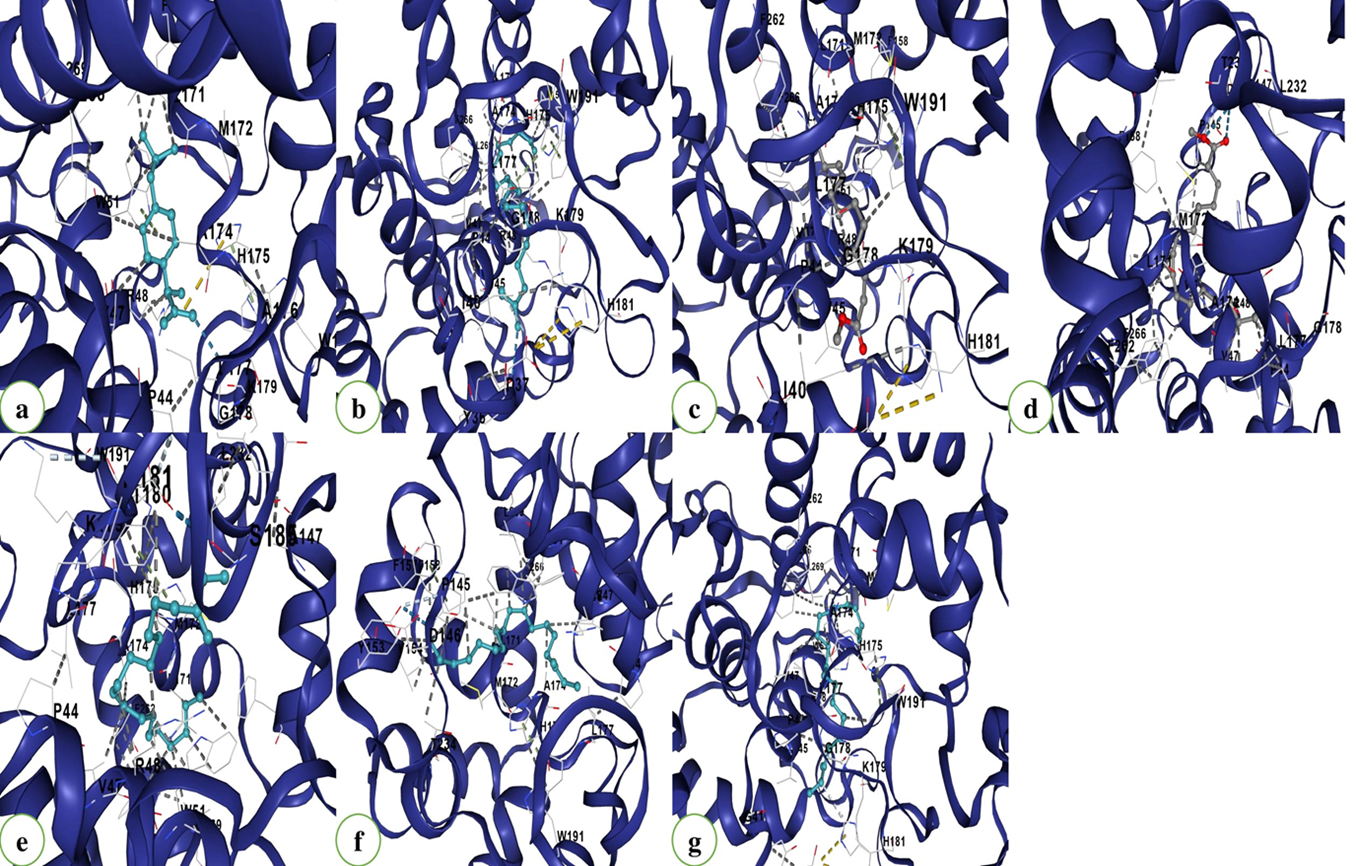
**Figure S4.** 3D binding interactions of 5-LOX with **(a)** Ibuprofen (standard drug); **(b)** Cyclopropaneoctanoic acid, 2-octyl-, methyl ester; **(c)** Linoleic acid, methyl ester; **(d)** Methyl 2-octylcyclopropene-1-heptanoate; **(e)** Oleic acid, methyl ester; **(f)** Palmitic acid, methyl ester; and **(g)** Stearic acid, methyl ester.

**
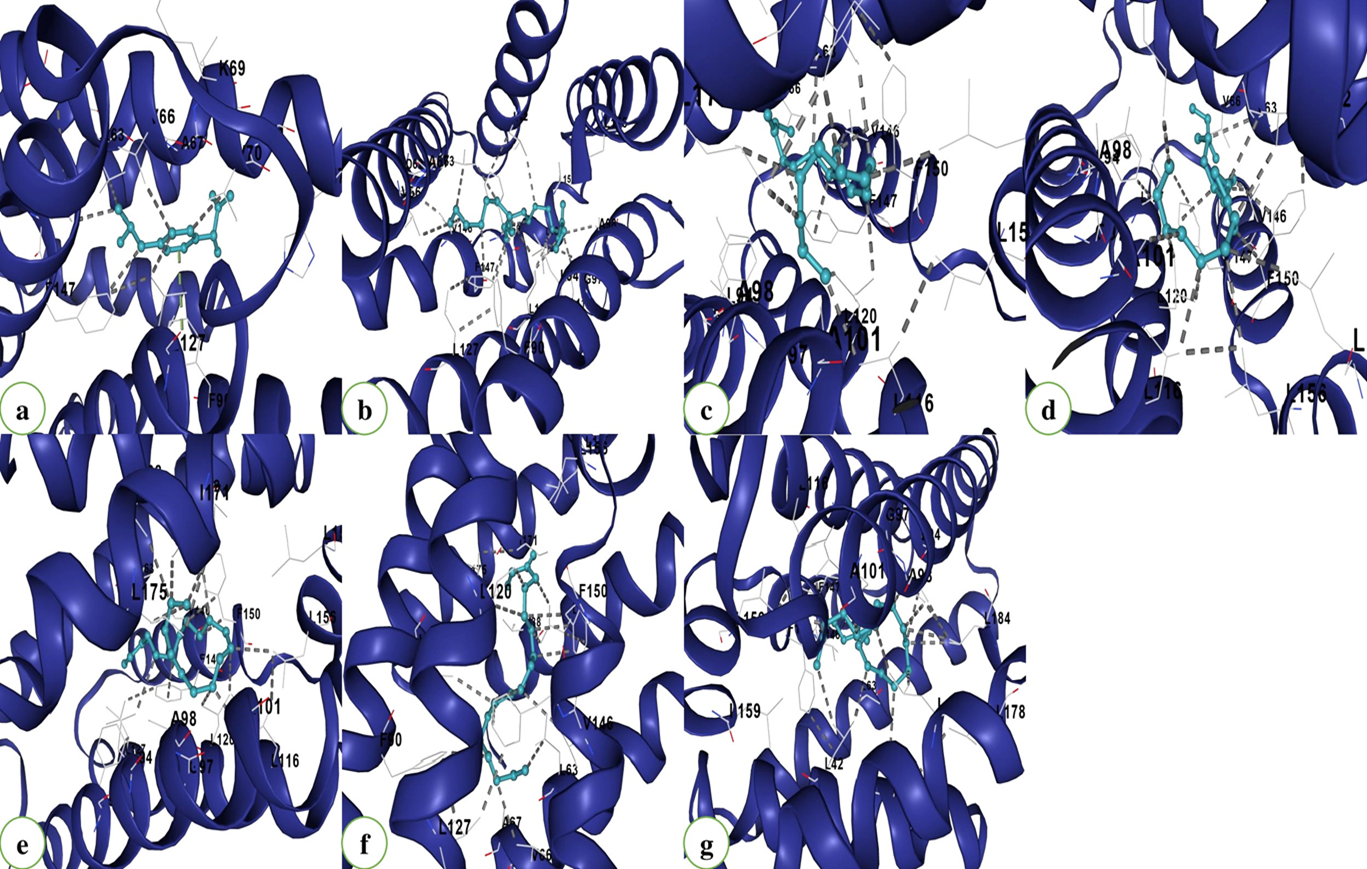
Figure S5.** 3D binding interactions of TNF-α with **(a)** Ibuprofen (standard drug); (b) Cyclopropaneoctanoic acid, 2-octyl-, methyl ester; **(c)** Linoleic acid, methyl ester; **(d)** Methyl 2-octylcyclopropene-1-heptanoate; **(e)** Oleic acid, methyl ester; **(f)** Palmitic acid, methyl ester; **(g)** Stearic acid, methyl ester.

**Table S1.** Molecular docking scores of free fatty acids against bacterial DNA gyrase (kcal/mol)

| Free Fatty Acid | Docking Scores (kcal/mol) | |
| --- | --- | --- |
|  | **6TTG (*S. aureus*)** | **1KZN (*E. coli*)** |
| Ciprofloxacin (Standard) | -7.8 | -7.8 |
| Linoleic acid | -5.6 | -5.7 |
| Oleic acid | -5.6 | -5.7 |
| Palmitic acid | -5.2 | -4.6 |
| Stearic acid | -4.9 | -5.6 |
| Cyclopropaneoctanoic acid, 2-octyl- | -5.6 | -6.0 |
| Methyl 2-octylcyclopropene-1-heptanoate (free acid form) | -5.2 | -5.2 |

**Note:** Free fatty acids generally showed improved binding affinity (ranging from 0.1 to 1.8 kcal/mol stronger) compared to their corresponding methyl esters (presented in main manuscript **Table 9 and Table 10**). The most pronounced improvements were observed for anti-inflammatory targets, with cyclopropaneoctanoic acid (free form) achieving -8.1 kcal/mol against 5-LOX and -7.8 kcal/mol against TNF-α, surpassing the reference drug ibuprofen (-7.9 and -6.5 kcal/mol, respectively).

**Table S2.** Molecular docking scores of free fatty acids against anti-inflammatory targets (kcal/mol)

| Free Fatty Acid | Docking Scores (kcal/mol) | | |
| --- | --- | --- | --- |
|  | **COX-2 (6COX)** | **5-LOX (1CYF)** | **TNF-α (3F4M)** |
| Ibuprofen (Standard) | -6.7 | -7.9 | -6.5 |
| Linoleic acid | **-7.2** | **-7.9** | -5.9 |
| Oleic acid | **-7.2** | -7.4 | -6.2 |
| Palmitic acid | -6.9 | -6.9 | **-6.7** |
| Stearic acid | **-7.1** | -7.5 | **-7.0** |
| Cyclopropaneoctanoic acid, 2-octyl- | **-7.6** | **-8.1** | **-7.8** |
| Methyl 2-octylcyclopropene-1-heptanoate (free acid form) | -6.7 | **-7.9** | **-7.8** |

**Note:** Cyclopropaneoctanoic acid (free form) achieved -8.1 kcal/mol against 5-LOX, surpassing ibuprofen (-7.9 kcal/mol).

**Table S3.** Redocking validation results

| **Protein Target** | **PDB ID** | **Co-crystallized Ligand** | **Docking Score (kcal/mol)** | **RMSD (Å)** | **Acceptable (<2.0 Å)** |
| --- | --- | --- | --- | --- | --- |
| ***S. aureus* DNA gyrase** | **6TTG** | **Ciprofloxacin** | -7.8 | 1.4 | Yes |
| ***E. coli* DNA gyrase** | **1KZN** | **Ciprofloxacin** | -7.8 | 1.6 | Yes |
| **COX-2** | **6COX** | **Ibuprofen** | -6.7 | 1.2 | Yes |
| **5-LOX** | **1CYF** | **Ibuprofen (co-crystallized analog)** | -7.9 | 1.8 | Yes |
| **TNF-α** | **3F4M** | **Ibuprofen** | -6.5 | 1.9 | Yes |

**Conclusion:** All RMSD values were ≤2.0 Å, confirming the accuracy and reproducibility of the docking protocol.

**Table S4.** Summary of key binding interactions for the most active compounds against all five protein targets

| Compound | Target | Key Hydrogen Bond Residues | Key Hydrophobic/van der Waals | Distance (Å) |
| --- | --- | --- | --- | --- |
| Linoleic acid methyl ester | 6TTG (*S. aureus* DNA gyrase) | N/A | ASP57, ARG84, GLY83, GLY85, PRO87, GLU58, ASN54, ASP81, SER55, THR173, ILE51, and LEU103 | 2.8 |
| Linoleic acid methyl ester | 1KZN (*E. coli* DNA gyrase) | ASN46, VAL120, SER121 | ALA96, HIS95, MET91, THR165, ASP73, ALA47, and ALU50. | 2.9 |
| Methyl 2-octylcyclopropene-1-heptanoate | 6COX (COX-2) | ARG44 | GLU46, GLY45, ASN39, GLN461, CYS36, GLN461, GLY135, ALA156, PRO154, LEU152, GLU465, ARG469, and ASN43. | 3.1 |
| Cyclopropaneoctanoic acid methyl ester | 1CYF (5-LOX) | LEU177 | MET172, HIS175, TRP191, GLY178, PRO44, VAL47, and PHE266. | 2.7 |
| Methyl 2-octylcyclopropene-1-heptanoate | 3F4M (TNF-α) | N/A | ALA67, VAL66, PHE90, LEU94, LEU159, LEU156, and GLY97. | 2.6 |


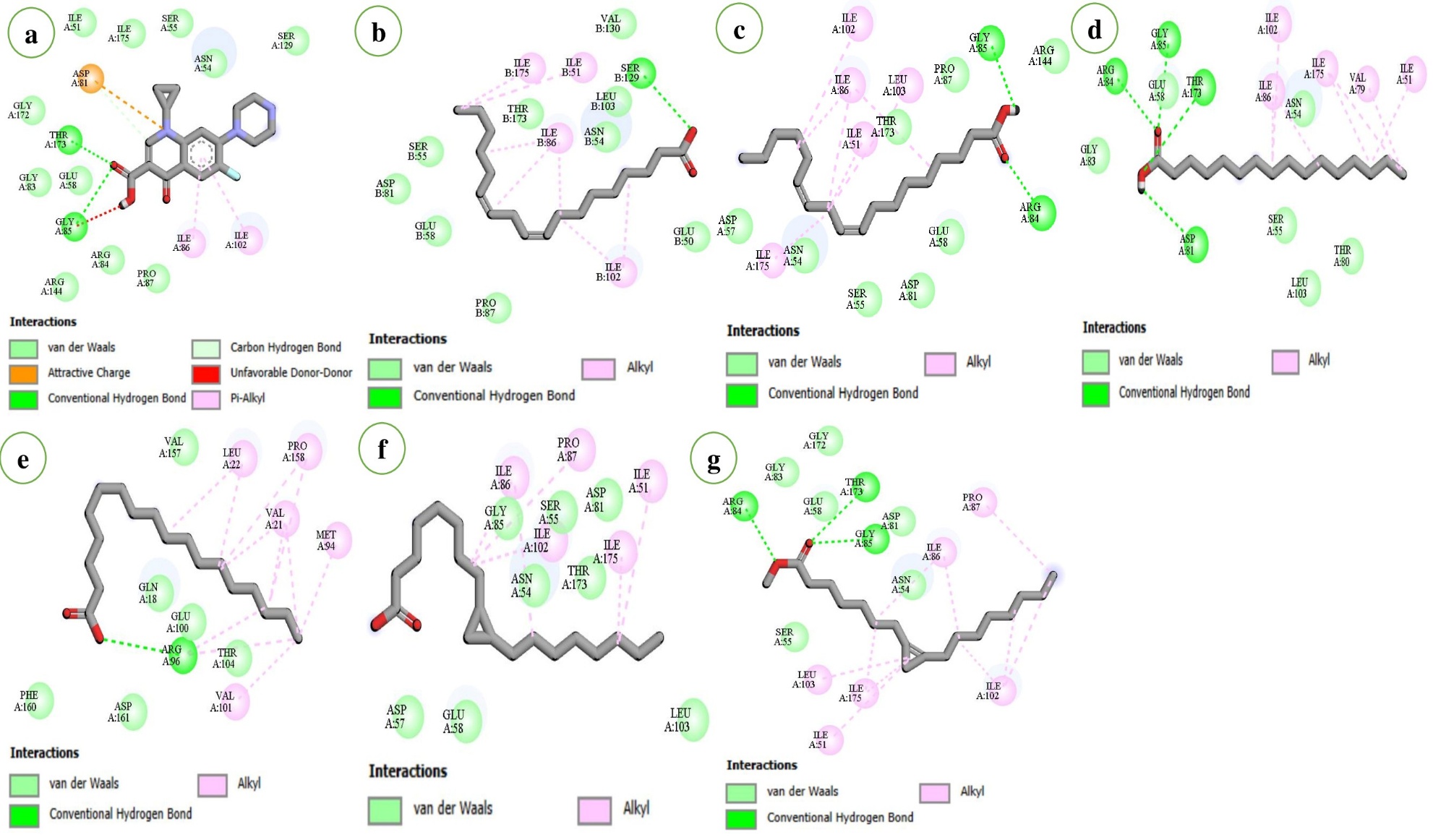
**Figure S6.** 2D binding interactions of DNA gyrase (6TTG) with **(a)** Ciprofloxacin (standard drug); **(b)** Linoleic acid; **(c)** Oleic acid; **(d)** Palmitic acid; **(e)** Stearic acid; **(f)** Cyclopropaneoctanoic acid, 2-octyl-; **(g)** Methyl 2-octylcyclopropene-1-heptanoate (free acid form).


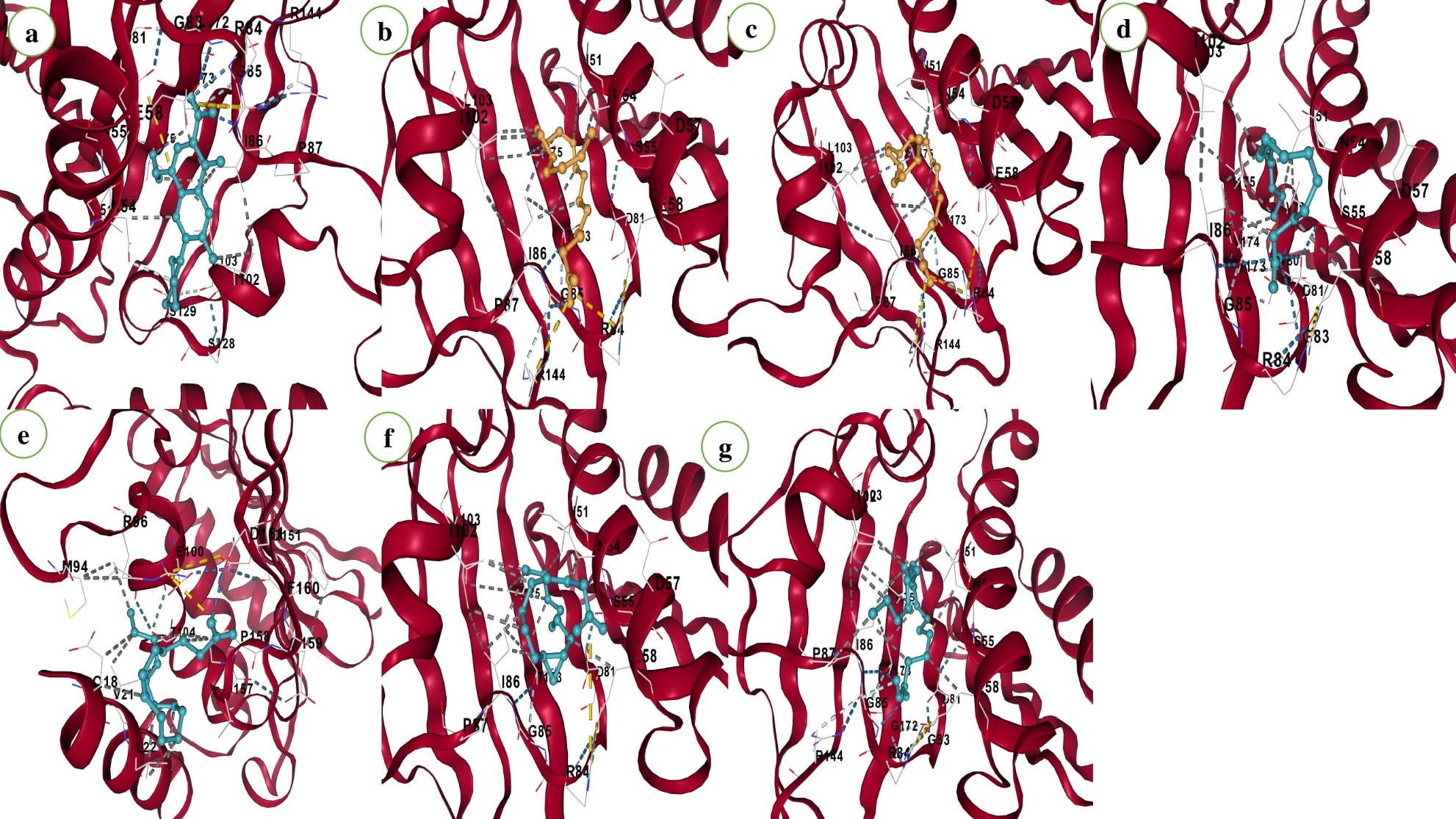


**Figure S7.** 3D binding interactions of DNA gyrase (6TTG) with **(a)** Ciprofloxacin (standard drug); **(b)** Linoleic acid; **(c)** Oleic acid; **(d)** Palmitic acid; **(e)** Stearic acid; **(f)** Cyclopropaneoctanoic acid, 2-octyl-; **(g)** Methyl 2-octylcyclopropene-1-heptanoate (free acid form).

**
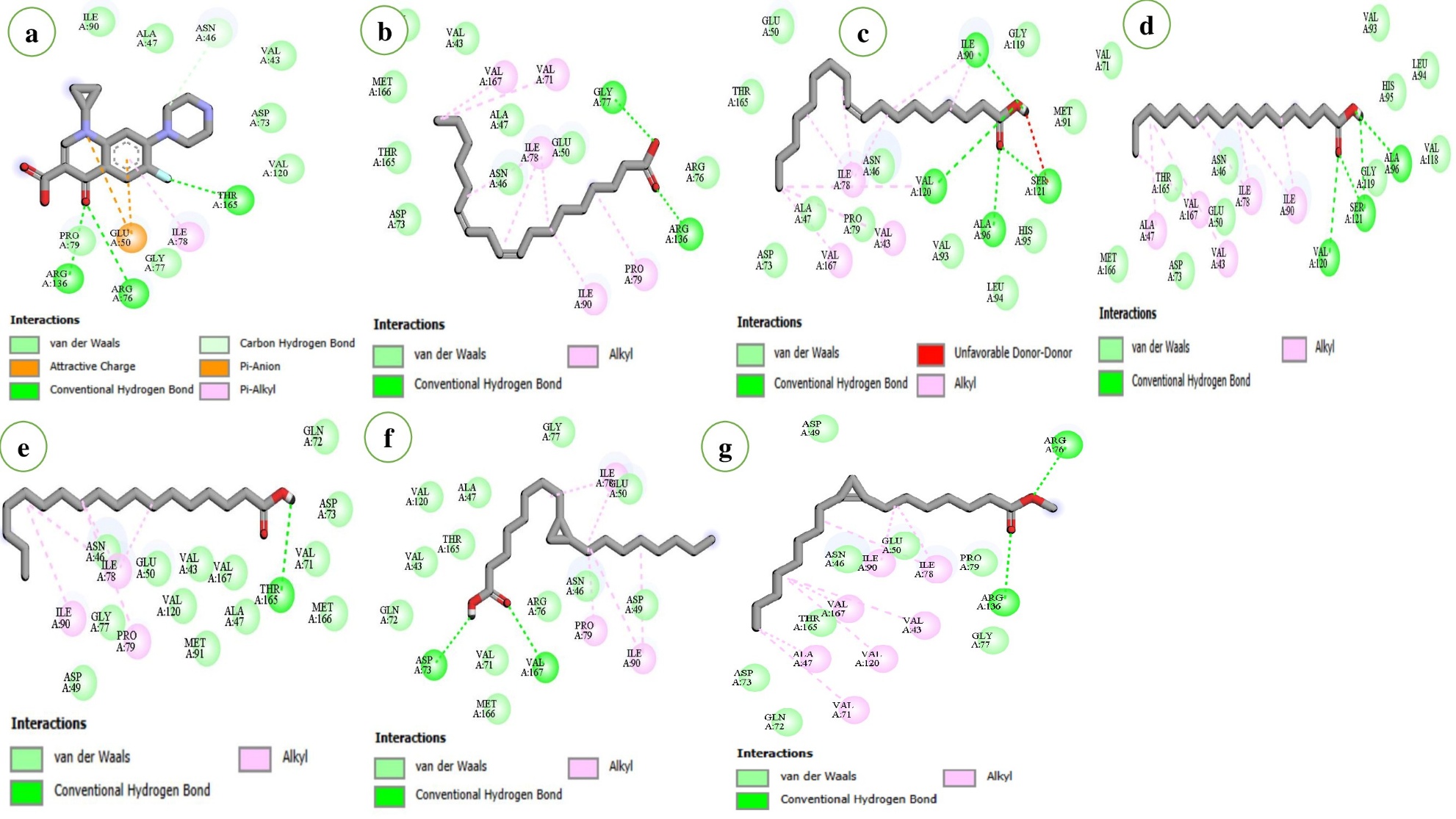
**

**Figure S8.** 2D binding interactions of DNA gyrase (1KZN) with **(a)** Ciprofloxacin (standard drug); **(b)** Linoleic acid; **(c)** Oleic acid; **(d)** Palmitic acid; **(e)** Stearic acid; **(f)** Cyclopropaneoctanoic acid, 2-octyl-; **(g)** Methyl 2-octylcyclopropene-1-heptanoate (free acid form).

**
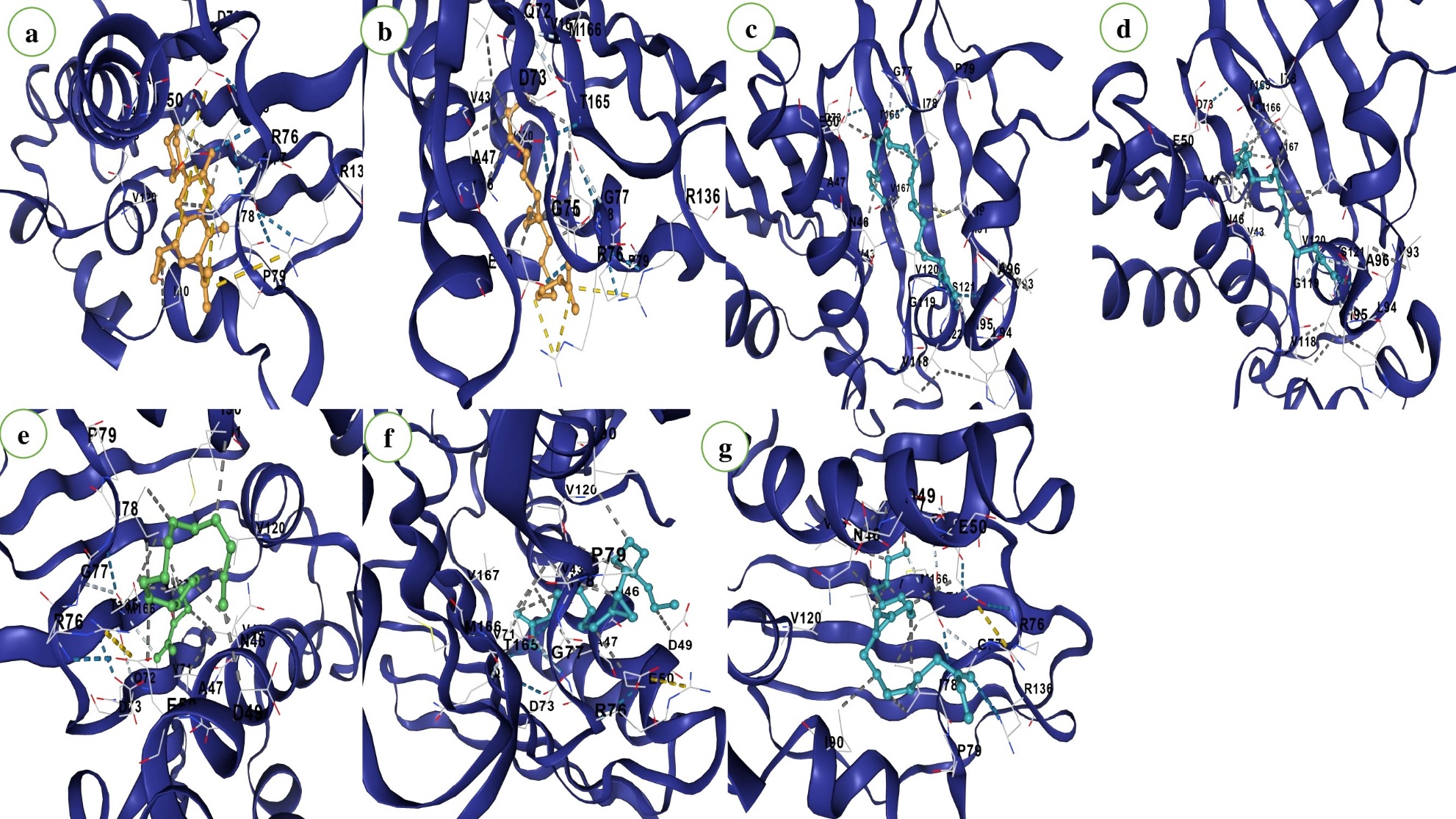
Figure S9.** 3D binding interactions of DNA gyrase (1KZN) with **(a)** Ciprofloxacin (standard drug); **(b)** Linoleic acid; **(c)** Oleic acid; **(d)** Palmitic acid; **(e)** Stearic acid; **(f)** Cyclopropaneoctanoic acid, 2-octyl-; **(g)** Methyl 2-octylcyclopropene-1-heptanoate (free acid form).

**
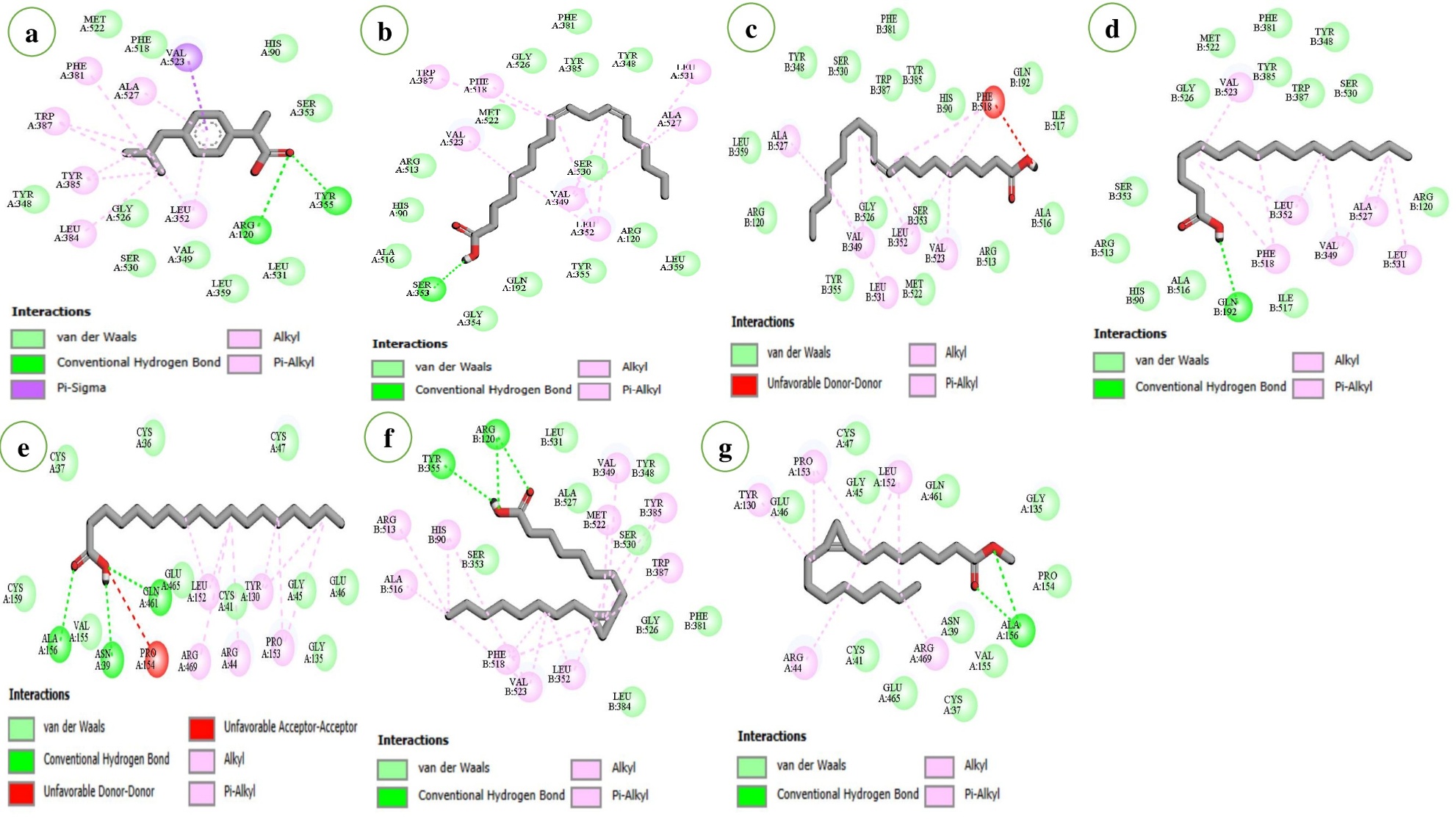
**

**Figure S10.** 2D binding interactions of COX-2 with **(a)** Ibuprofen (standard drug); **(b)** Linoleic acid; **(c)** Oleic acid; **(d)** Palmitic acid; **(e)** Stearic acid; **(f)** Cyclopropaneoctanoic acid, 2-octyl-; **(g)** Methyl 2-octylcyclopropene-1-heptanoate (free acid form).

**
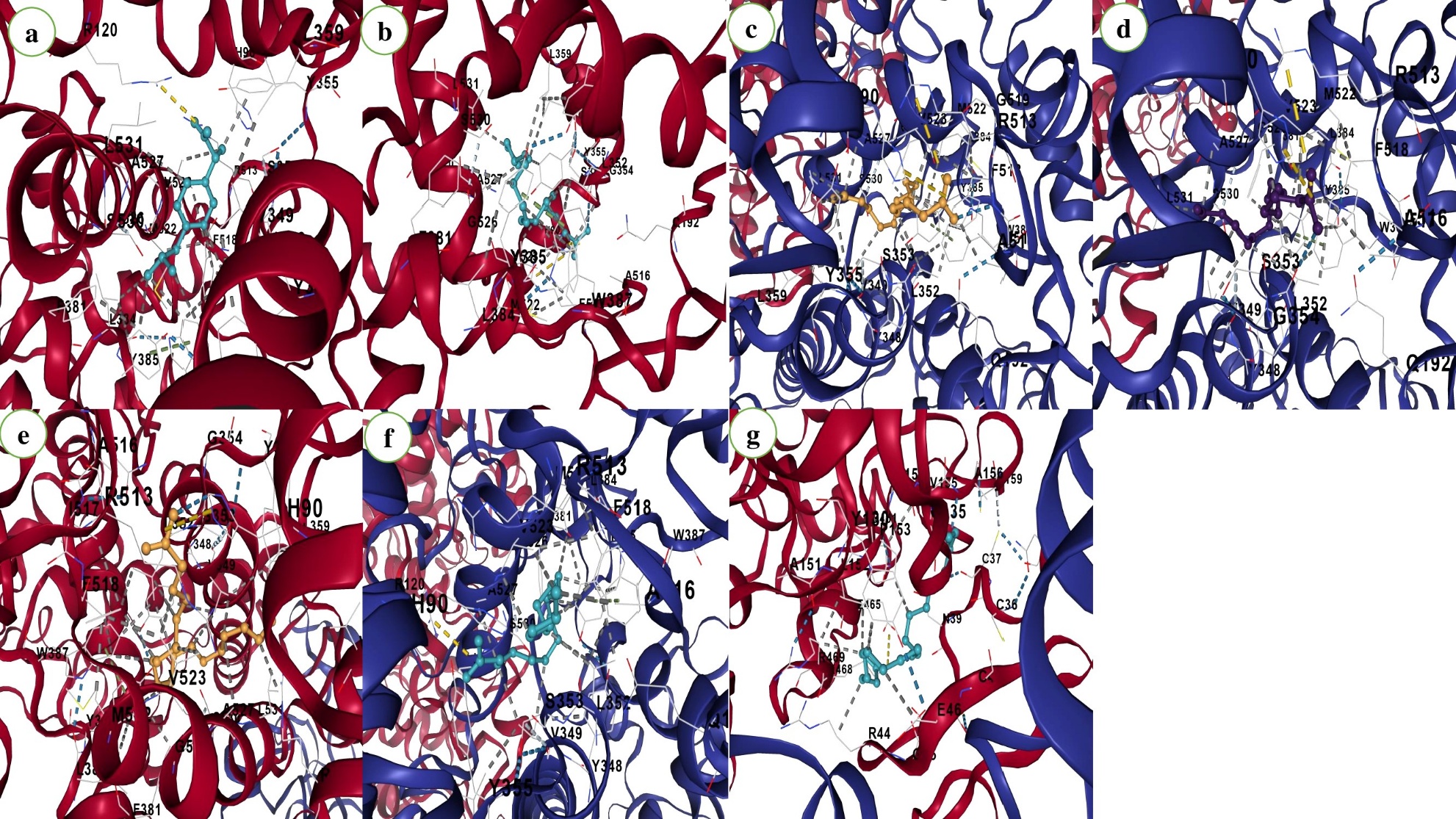
**

**Figure S11.** 3D binding interactions of COX-2 with **(a)** Ibuprofen (standard drug); **(b)** Linoleic acid; **(c)** Oleic acid; **(d)** Palmitic acid; **(e)** Stearic acid; **(f)** Cyclopropaneoctanoic acid, 2-octyl-; **(g)** Methyl 2-octylcyclopropene-1-heptanoate (free acid form).

**
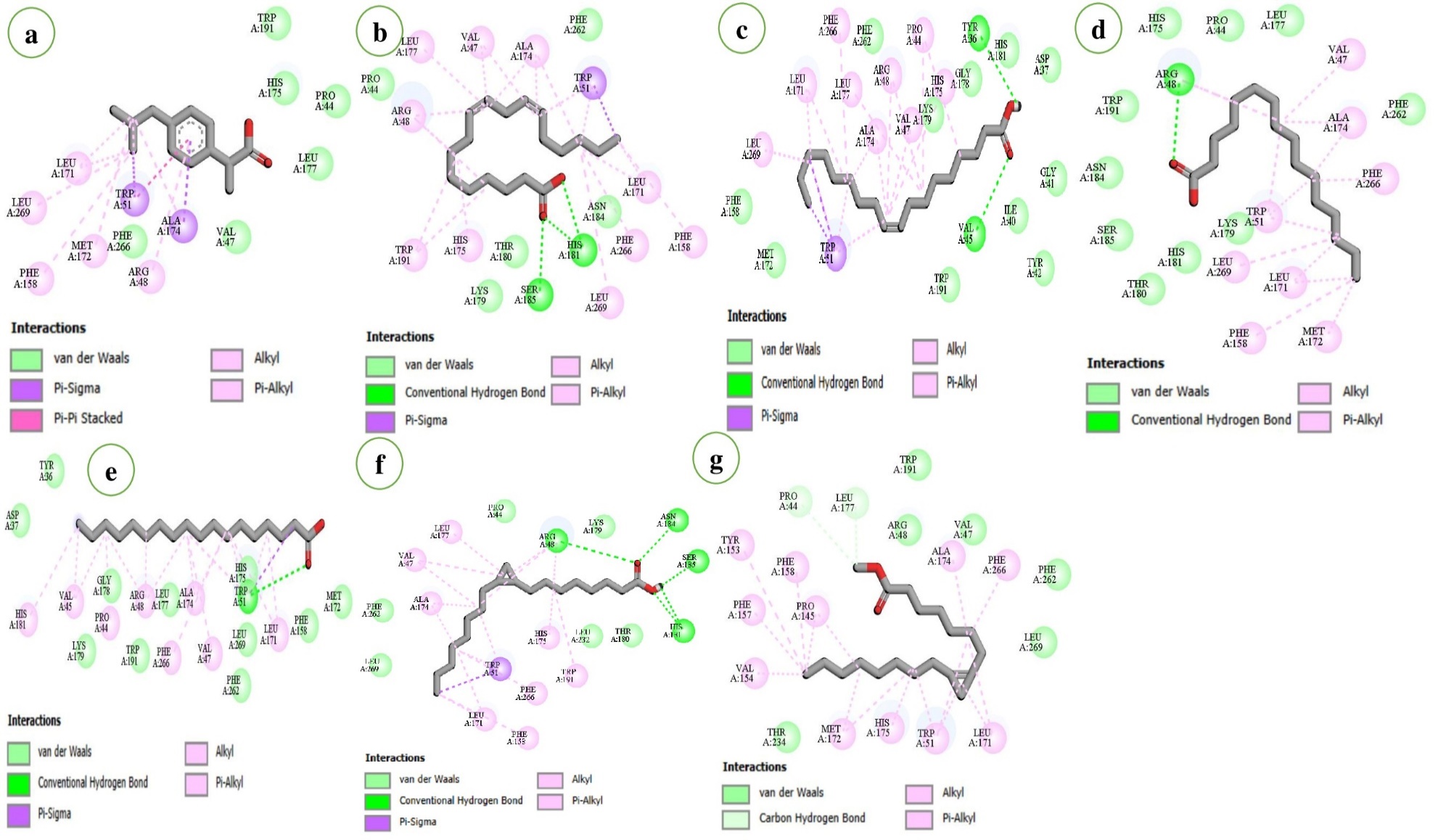
**

**Figure S12:** 2D binding interactions of 5-LOX with **(a)** Ibuprofen (standard drug); **(b)** Linoleic acid; **(c)** Oleic acid; **(d)** Palmitic acid; **(e)** Stearic acid; **(f)** Cyclopropaneoctanoic acid, 2-octyl-; **(g)** Methyl 2-octylcyclopropene-1-heptanoate (free acid form).

**
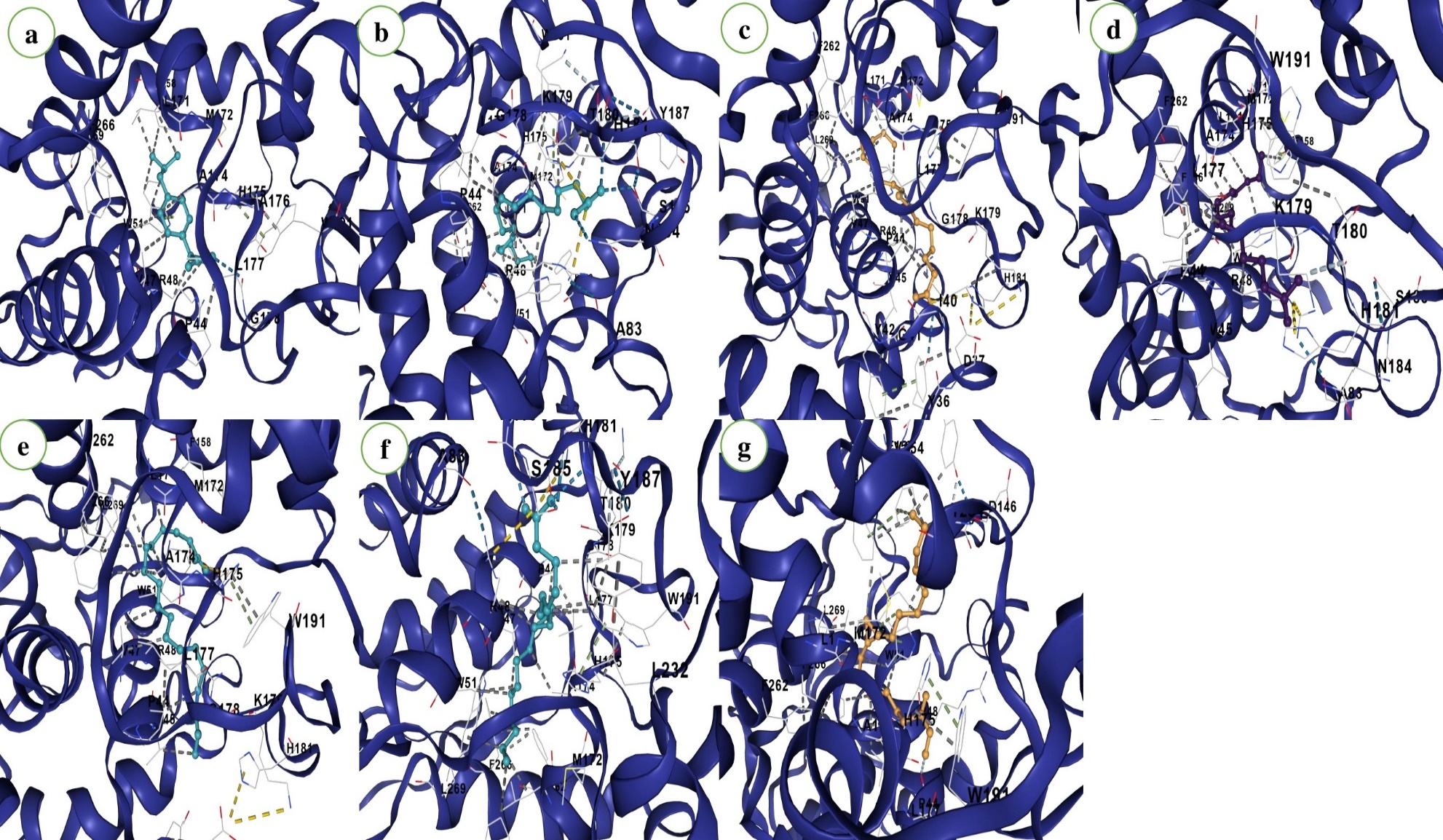
**

**Figure S13:** 3D binding interactions of 5-LOX with **(a)** Ibuprofen (standard drug); **(b)** Linoleic acid; **(c)** Oleic acid; **(d)** Palmitic acid; **(e)** Stearic acid; **(f)** Cyclopropaneoctanoic acid, 2-octyl-; **(g)** Methyl 2-octylcyclopropene-1-heptanoate (free acid form).

**
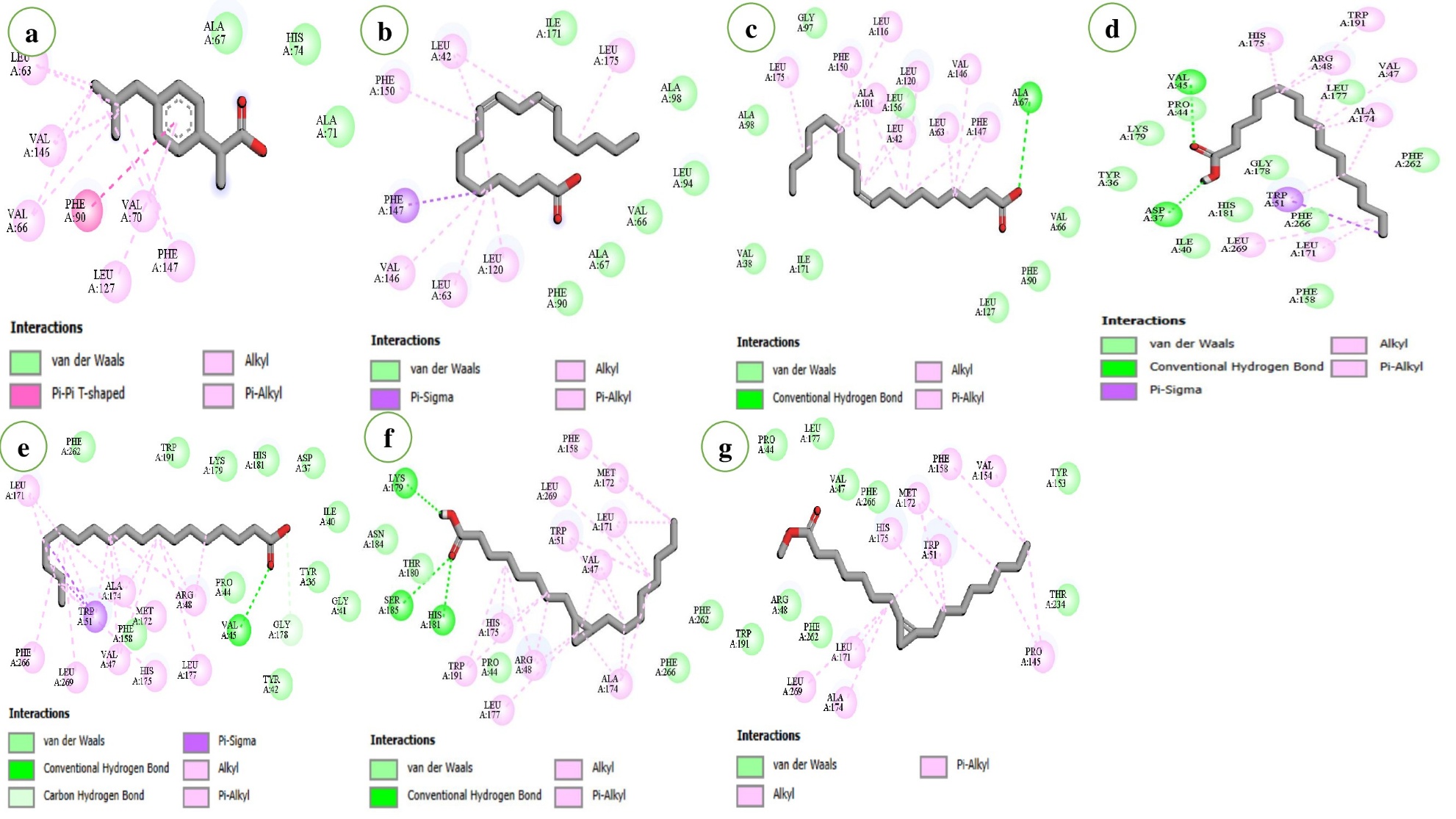
**

**Figure S14:** 2D binding interactions of TNF-α with **(a)** Ibuprofen (standard drug); **(b)** Linoleic acid; **(c)** Oleic acid; **(d)** Palmitic acid; **(e)** Stearic acid; **(f)** Cyclopropaneoctanoic acid, 2-octyl-; **(g)** Methyl 2-octylcyclopropene-1-heptanoate (free acid form).

**
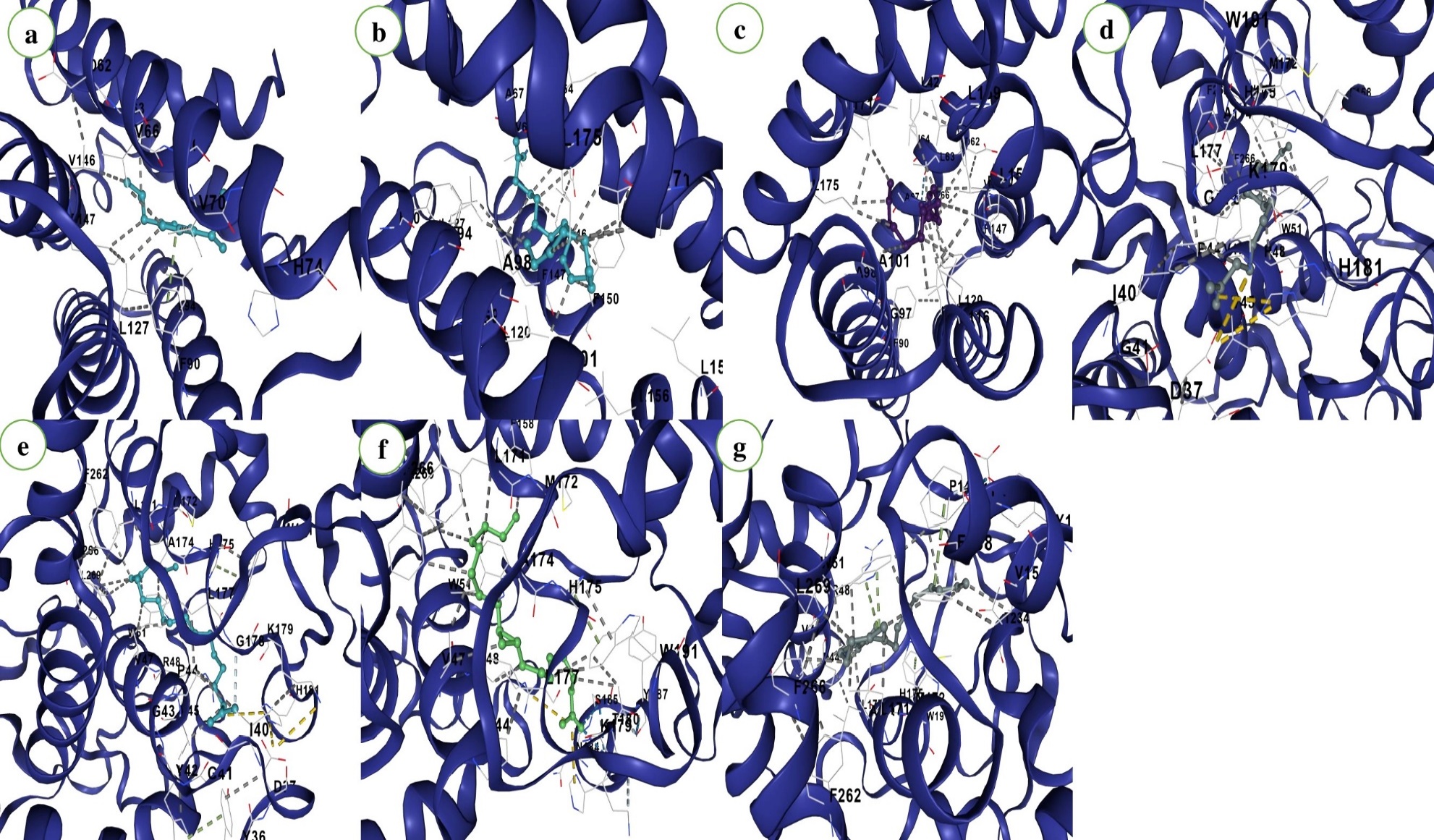
**

**Figure S15:** 3D binding interactions of TNF-α with **(a)** Ibuprofen (standard drug); **(b)** Linoleic acid; **(c)** Oleic acid; **(d)** Palmitic acid; **(e)** Stearic acid; **(f)** Cyclopropaneoctanoic acid, 2-octyl-; **(g)** Methyl 2-octylcyclopropene-1-heptanoate (free acid form).
